# Integrated Genomics Identifies miR-181/TFAM Pathway as a Critical Driver of Drug Resistance in Melanoma

**DOI:** 10.3390/ijms22041801

**Published:** 2021-02-11

**Authors:** Anna Barbato, Antonella Iuliano, Mariagrazia Volpe, Romina D’Alterio, Simona Brillante, Filomena Massa, Rossella De Cegli, Sabrina Carrella, Massimiliano Salati, Annapina Russo, Giulia Russo, Sara Riccardo, Davide Cacchiarelli, Mariaelena Capone, Gabriele Madonna, Paolo A. Ascierto, Brunella Franco, Alessia Indrieri, Pietro Carotenuto

**Affiliations:** 1 TIGEM, Telethon Institute of Genetics and Medicine, 80078 Naples, Italy; anna.barbato@tigem.it (A.B.); a.iuliano@tigem.it (A.I.); m.volpe@tigem.it (M.V.); r.dalterio@tigem.it (R.D.); s.brillante@tigem.it (S.B.); f.massa@tigem.it (F.M.); decegli@tigem.it (R.D.C.); carrella@tigem.it (S.C.); d.cacchiarelli@tigem.it (D.C.); franco@tigem.it (B.F.); indrieri@tigem.it (A.I.); 2 Medical Oncology Unit, University Hospital of Modena, Modena Cancer Centre, 41100 Modena, Italy; massisalati@gmail.com; 3 Department of Pharmacy, University of Naples “Federico II”, 80131 Naples, Italy; annapina.russo@unina.it (A.R.); giulia.russo@unina.it (G.R.); 4 Next Generation Diagnostic srl, 80078 Pozzuoli, Naples, Italy; sara.riccardo@ngdx.eu; 5 Armenise/Harvard Laboratory of Integrative Genomics, 80078 Naples, Italy; 6 Medical Genetics, Department of Translational Medical Science, University of Naples “Federico II”, 80131 Naples, Italy; 7 Melanoma, Cancer Immunotherapy and Development Therapeutics Unit, Istituto Nazionale Tumori IRCCS Fondazione “G. Pascale”, 80131 Naples, Italy; marilenacapone@gmail.com (M.C.); gabriele.madonna@yahoo.it (G.M.); paolo.ascierto@gmail.com (P.A.A.); 8 Institute for Genetic and Biomedical Research (IRGB), National Research Council (CNR), 20090 Milan, Italy

**Keywords:** miR-181, melanoma, mitochondria, TFAM, microRNA, target therapy, cancer resistance, BRAF inhibitors, Dabrafenib, biomarkers

## Abstract

MicroRNAs (miRNAs) are attractive therapeutic targets and promising candidates as molecular biomarkers for various therapy-resistant tumors. However, the association between miRNAs and drug resistance in melanoma remains to be elucidated. We used an integrative genomic analysis to comprehensively study the miRNA expression profiles of drug-resistant melanoma patients and cell lines. MicroRNA-181a and -181b (miR181a/b) were identified as the most significantly down-regulated miRNAs in resistant melanoma patients and cell lines. Re-establishment of miR-181a/b expression reverses the resistance of melanoma cells to the BRAF inhibitor dabrafenib. Introduction of miR-181 mimics markedly decreases the expression of TFAM in A375 melanoma cells resistant to BRAF inhibitors. Furthermore, melanoma growth was inhibited in A375 and M14 resistant melanoma cells transfected with miR-181a/b mimics, while miR-181a/b depletion enhanced resistance in sensitive cell lines. Collectively, our study demonstrated that miR-181a/b could reverse the resistance to BRAF inhibitors in dabrafenib resistant melanoma cell lines. In addition, miR-181a and -181b are strongly down-regulated in tumor samples from patients before and after the development of resistance to targeted therapies. Finally, melanoma tissues with high miR-181a and -181b expression presented favorable outcomes in terms of Progression Free Survival, suggesting that miR-181 is a clinically relevant candidate for therapeutic development or biomarker-based therapy selection.

## 1. Introduction

Melanoma represents one of the most aggressive skin cancers with a significantly increased incidence in recent decades [1,2,3]. Current therapeutic strategies include surgical intervention, chemotherapy, targeted- and immune-therapies. In the last few years, molecular targeted- and immuno-therapies have provided marked improvements in terms of patient survival [4]. In particular, the BRAF and MEK inhibitors currently in use, such as dabrafenib, vemurafenib and trametinib, showed encouraging response rates but their efficacy is limited by mechanisms of intrinsic or acquired resistance, frequently occurring in melanoma [5]. Melanoma cells are characterized by high heterogeneity and phenotypic plasticity which allow them to re-activate oncogenic processes, such as the MAPK pathway and thus sustain drug-resistance [5,6]. The introduction of immune-therapies and their combination with BRAF inhibitors (BRAFi) and MEK inhibitors (MEKi) has provided significant and more durable clinical response but the majority of patients do not respond to immunotherapy or inevitably develop resistance to drugs after a period of treatment [5,6,7]. The observations described above highlighted two unmet clinical needs: the first is the development of novel therapeutic strategies able to overcome resistance and the second is the identification of biomarkers able to monitor and/or predict the response to therapies.

Epigenetic and/or post-transcriptional mechanisms were identified as key molecular-regulators of drug-resistance in melanoma [8]. In this context, non-coding RNAs, represent an attractive molecular class for their capacity to regulate the expression of genes and proteins involved in resistance-related pathways [8]. In recent years several studies have analyzed the involvement of microRNAs (miRNAs), a class of small non-coding RNAs in molecular mechanisms orchestrating melanoma growth, survival, invasion and drug-resistance [8,9]. Additionally, recent studies have uncovered that aberrantly expressed miRNAs are implicated in tumor development, as well as being attractive candidate biomarkers and potential targets for therapy [10,11,12].

The miR-181 family belongs to a conserved group of miRNAs regulating many relevant biological processes such as cell proliferation, apoptosis, autophagy, mitochondrial function and immune response [13]. In the human genome, the precursors of miR-181a and -181b (pre-mir-181a1b1 and pre-mir-181a2b2) are located in two genetic clusters on chromosomes 1 and 9 [13]. The pre-mir-181a1b1 and pre-mir-181a2b2 give rise to two identical copies of mature miR-181a or miR-181b. Notably, miR-181a or miR-181b (miR181a/b) share the same seed region and are therefore predicted to recognize a similar set of target genes [13]. Several studies have demonstrated that miR-181 family members have a pivotal role in cancer and in establishment of drug resistance [13,14,15,16]. In fact, accumulating evidence showed that miR-181a and -181b (miR181a/b) are able to modulate a wide range of mRNA targets belonging to the main cancer-related pathways, including RAS, cyclin D, C-MYC, E2F, BCL2 and cytochrome C [13,14,15,16]. However, little is known regarding the potential role of miR-181a/b in melanoma resistance to BRAFi.

In this paper, a comprehensively study of the miRNA expression profiles of drug-resistant melanoma patients and cell lines was conducted, thus identifying miR-181a/b as significantly down-regulated miRNAs in resistant melanoma patients and cell lines. Our study revealed the potential involvement of miR-181a/b in BRAFi resistant melanoma and raised the possibility that this might be exploited therapeutically. Indeed, re-establishment of miR-181a/b expression increased sensitivity to BRAFi or counteracted its growth stimulating effect. Finally, we found that high levels of miR-181a/b were associated with a favorable clinical response in melanoma patients treated with BRAFi or BRAFi plus MEKi. We showed that the miR-181a/b function is determined by direct binding to their target *TFAM* (Mitochondrial transcription factor A). Overexpression of miR-181a/b or inhibition of *TFAM* expression may thereby represent novel therapeutic approaches to increase the efficacy of BRAFi. Moreover, we propose that miR-181a/b expression levels may be a useful biomarker to improve patient selection for targeted therapy and for monitoring the onset of resistance.

## 2. Results

### 2.1. Melanoma Cells with Acquired Resistance to Dabrafenib Display Changes in miRNA Expression Pattern

In order to identify miRNAs potentially involved in BRAF inhibitor resistance, we analyzed the miRNAome of melanoma cell lines sensitive to the BRAF inhibitor dabrafenib and their resistant counterparts.

To explore this experimentally, we generated BRAF inhibitor-resistant A375 and M14 cells (A375-BIR and M14-BIR) by continuous selective culture.

**Figure 1:**
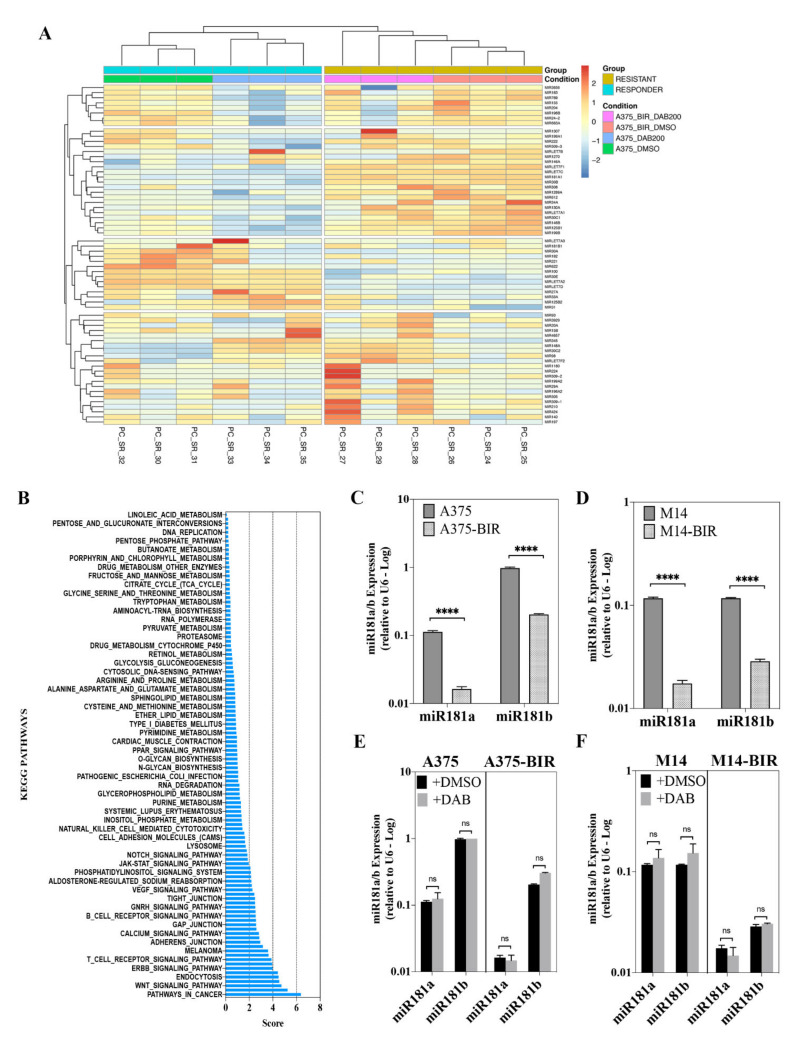
(**A**) Hierarchical clustering analysis and heat map based on the expression profiles of 65 miRNAs in responding and non-responding melanoma cells to dabrafenib treatment. Cluster analysis grouped samples and miRNAs according to similarity in expression. miRNAs are in rows and samples in columns. The miRNA clustering tree is indicated on the left and the sample clustering tree is at the top. Red color represents up-regulated expression and blue marks downregulated genes. Yellow indicates resistant cells and blue indicates responsive cells. miRNA, microRNA. To create the heatmap we converted the read counts into log2-counts-per-million (logCPM) values. (**B**) Putative target genes of differentially expressed miRNAs were obtained from TargetScan and used for KEGG pathway enrichment analysis. Only pathways with an adjusted *p* value < 0.01 were considered and listed according to a decreasing value of the combined score. (**C**,**D**) The expression levels of miR-181a and b in A375, A375-BIR, M14 and M14-BIR melanoma cells, measured by quantitative Real-Time PCR (qRT-PCR). (**E**,**F**) The expression levels of miR-181a and b in A375, A375-BIR, M14 and M14-BIR melanoma cells, treated with 200 nM dabrafenib (DAB) and vehicle control (DMSO), measured by qRT-PCR. The miRNA expression levels were normalized to the internal control U6. **** *p* < 0.0001; ns, not significantly.

Using Small-RNAseq, a microRNA differential expression (DE) analysis on A375 and A375-BIR cells was performed to identify miRNAs regulating the resistance to dabrafenib treatment. Sixty-five miRNAs were found to have 1.5-fold or greater differences in levels in A375 cells in comparison to the resistant counterpart (Figure 1A and Table1) in both control (0.1% DMSO) and dabrafenib treated groups. The majority of differentially expressed miRNAs showed high-fold changes (greater than 2). Data are presented in Figure 1A as a heatmap showing differentially expressed (DE) miRNAs up-regulated (n = 27) and down-regulated (n = 38) in A375 sensitive cells with respect to A375-BIR.

The analysis of DE miRNAs allowed us to identify several miRNAs (e.g., in down-regulated group miR-126-3p, miR-146a-5p, miR-224-5p; in up-regulated group miR-424-3p, miR-503-5p, miR-509-3p) known to be involved in the onset and/or progression of melanoma and in resistance, thus confirming the quality of our approach and results [17,18,19,20,21] (Table 1).

A functional annotation analysis was next performed on predicted target mRNAs of the sixty-five DE miRNAs for the enrichment of KEGG pathways. Several pathways previously involved in resistance to BRAFi and/or regulation of melanoma growth and progression were found to be enriched, including MAP kinase-, PI3K/AKT-, ErbB-, Hippo-, WNT-, focal adhesion- and a large series of metabolism-related signaling pathways (Figure 1B).

Within the DE miRNA group, we also identified miRNAs previously associated with drug resistance, including miR-181a/b [13,15,22,23,24], whose role in melanoma and resistance to target-therapy had not been previously thoroughly investigated. In order to study novel miRNAs regulating sensitivity and/or resistance to BRAF inhibitors, we focused our attention on miR-181a/b. Despite several studies which showed their involvement in cancer progression and resistance, the role of miR-181a/b was not previously addressed in melanoma.

Quantitative Real-Time PCR (RT-PCR) results confirmed that mature forms of miR-181a/b were significantly up-regulated in two melanoma dabrafenib-sensitive cells (A375, M14) with respect to resistant lines (A375-BIR, M14-BIR) (Figure 1C,D). To validate the sensitivity and the appropriateness of the Small-RNAseq approach we also analyzed the expression of miR-224-5p in A375 and A375-BIR cells. Our results indicate that the expression of this microRNA was significantly down-regulated in resistant cell lines (A375-BIR) (Supplementary Materials Figure S1A). Moreover, expression analysis of miR-181a/b revealed no significant differences between dabrafenib- and control-treated sensitive and resistant cells (Figure 1E,F), thus suggesting that the expression of miR-181a/b is not affected by dabrafenib treatment. On the contrary, the expression of miR-224-5p was strongly induced by dabrafenib treatment (Supplementary Materials Figure S1A). Furthermore, the expression of miR-181a/b is not modulated by other stress-inducing drugs, as revealed by RT-PCR on A375 melanoma cells treated with actinomycin D (Supplementary Materials Figure S1B), providing evidence that their role is not functionally associated with adaptive responses to drugs. Taken together the above described findings provided new results in exploring the miRNAome of sensitive and target therapy-resistant melanoma cells and allowed us to identify miRNA-181a and -181b as new potential modulators of melanoma resistance to BRAF inhibitors.

### 2.2. miR-181a/b Replacement Affects the Growth of Melanoma Cells Sensitive and Resistant Cells to Dabrafenib

**Figure 2:**
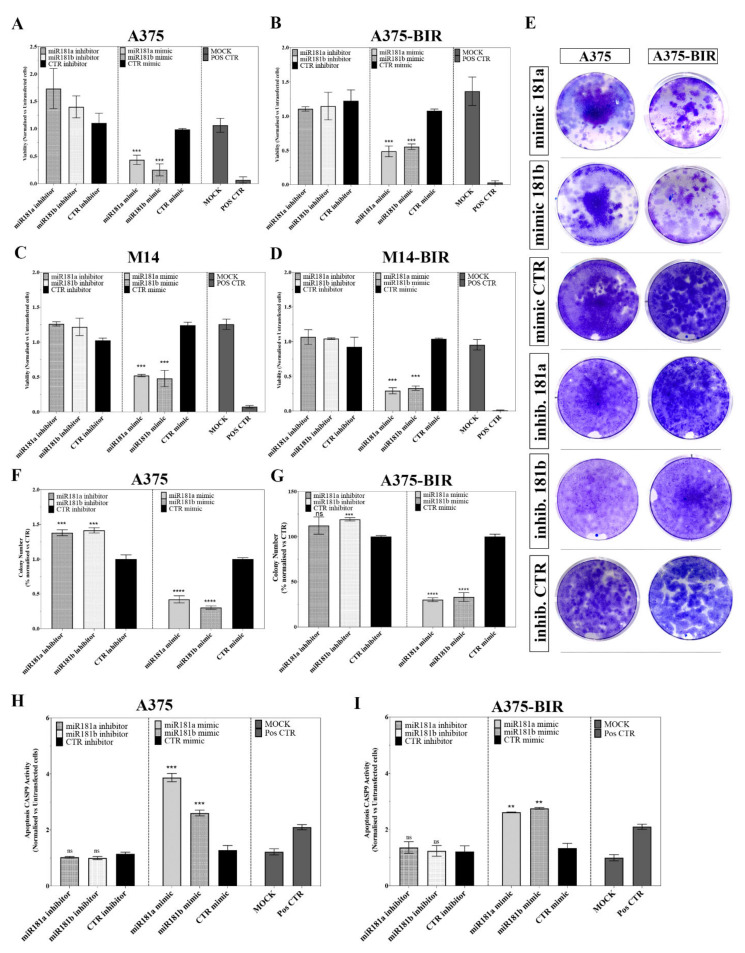
(**A,B**) A375 and A375-BIR cells were transiently transfected with 40 nM of the miR-181a and -181b mimic or inhibitor or the appropriate controls and assayed for proliferation by the MTT viability assay. Data are expressed in terms of percentage of cell viability as compared to untransfected cells. Each value represents the arithmetic mean of three independent experiments performed with triplicate samples. *** *p* < 0.001. (**C**,**D**) M14 and M14-BIR cells were transiently transfected with 40 nM of the miR-181a and -181b mimic or inhibitor or the appropriate controls and assayed for proliferation by the MTT assay. Data are expressed in terms of percentage of cell viability as compared to untransfected cells. Each value represents the arithmetic mean of three independent experiments performed with triplicate samples. *** *p* < 0.001. (**E**–**G**) Colony formation assays were performed to determine the colony formation ability of A375 and A375-BIR melanoma cells transfected with miR-181a and -b mimics or inhibitor or the appropriate controls. Representative images and quantification of visible colonies have been presented. The colony number in the DMSO-treated group was set as 100%. All the experiments were performed in triplicate and the relative colony formation rates are shown as the mean +/− SD. *** *p* < 0.001; **** *p* < 0.0001; ns, not significant. **H**–**I)** Effect of miRNA-181a and b forced expression or inhibition on CASP-9 activation in A375 and A375-BIR cells. The determination of CASP-9 activity was carried out by using Caspase-Glo^®^ 9 assay. Data are expressed in fold change relative to vehicle control +/− SD of three independent assays with 3 replicates for each one. *** *p* < 0.001 and ** *p* < 0.01 versus vehicle control; ns, not significant.

We next investigated whether replacement or inhibition of miR-181a/b could affect the proliferation of two melanoma cell lines sensitive (A375, M14) or resistant to BRAF inhibitors (A375-BIR, M14-BIR). As illustrated in Figure 2A–D, transfection of oligonucleotides mimicking the mature forms of miR-181a/b significantly suppressed the viability of both sensitive (Figure 2A–C) and resistant (Figure 2B–D) cells compared to controls. Using a similar approach, we also examined the effect of inhibition of endogenous miR-181a/b activity on cell viability. In sensitive cells, A375 and M14, the inhibition of the two miRNAs enhanced cell proliferation with respect to the controls (Figure 2A–C), while no significant results were obtained in both resistant cell lines (Figure 2B–D).

We next evaluated the effect of miR-181a/b overexpression on the colony formation abilities of melanoma cells. Results clearly demonstrated that forced expression of the two miRNAs significantly reduced the number of colonies in both A375 resistant and parental cell lines (Figure 2E–G). Moreover, the transfection of oligonucleotides inhibiting the expression of miR-181a/b increased the colony counts in both cell type, thus indicating that miR-181a/b depletion may trigger tumor growth *in vitro* (Figure 2E–G). The transfection efficiency of miR-181a/b in A375 and A375-BIR cells was evaluated by qRT-PCR (Supplementary Materials Figure S1C). These results suggest that miR-181a and -181b are effective tumor suppressor microRNAs and their replacement in BRAF inhibitor resistant cell lines (A375-BIR, M14-BIR) clearly inhibits cell growth.

We further investigated if miR-181a/b may suppress tumor growth in vitro by promoting apoptosis. Biochemical assays measuring caspase 9 (CASP-9) activity showed that forced expression of miR-181a/b significantly increased CASP-9 activity both in sensitive and in resistant melanoma cells compared to controls (Figure 2H,I). On the other hand, miR-181a/b silencing resulted in no significant modulation on apoptosis (Figure 2H,I). The findings described above suggest that miR-181a/b may act as tumor suppressor in melanoma cell lines by inhibiting tumor proliferation and activating apoptosis.

### 2.3. miR-181a/b Replacement Sensitizes Resistant Cells to Dabrafenib

Having demonstrated that miR-181a/b were down-regulated in dabrafenib-resistant cells and that their replacement impaired cell proliferation, we investigated whether re-expression of both microRNAs could also sensitize melanoma cells to dabrafenib and thus attenuate resistance.

**Figure 3:**
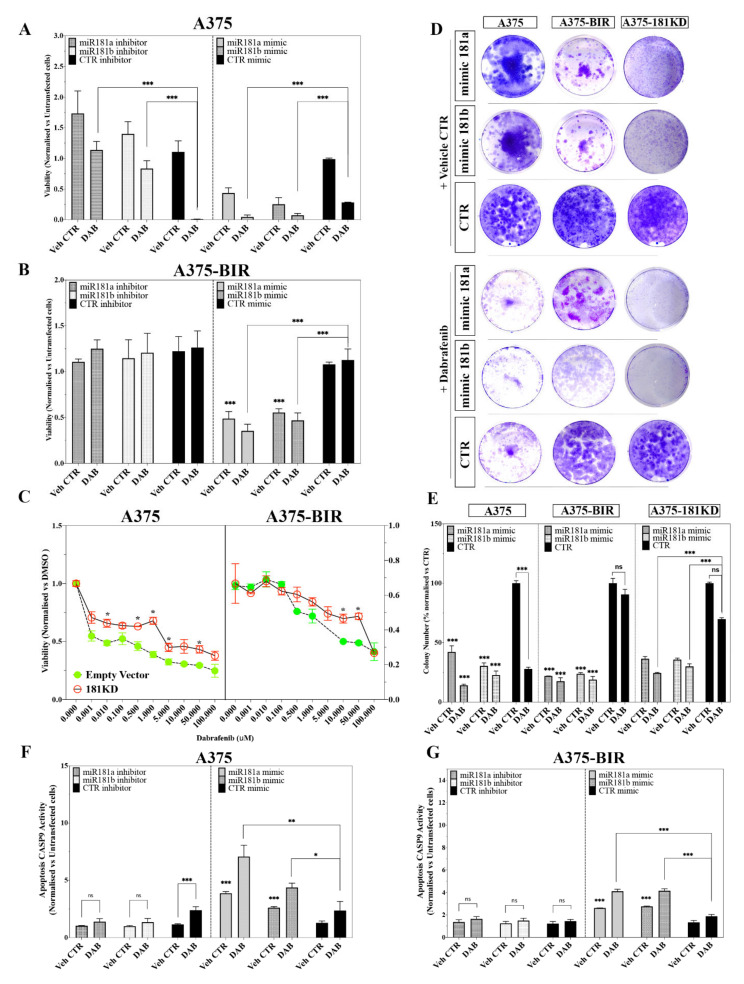
(**A**,**B**) A375 (**A**) and A375-BIR (**B**) cells transiently transfected with 40 nM of the miR-181a and -181b mimic or inhibitor or the appropriate controls were treated with 200 nM dabrafenib and assayed for proliferation by the MTT assay. Data are expressed in terms of percentage of cell viability as compared to untransfected cells. Each value represents the arithmetic mean of three independent experiments performed with triplicate samples. *** *p* < 0.001; * *p* < 0.01. (**C**) Effect of miR-181a/b knockdown on cell proliferation in A375 and A375-BIR melanoma cells treated with increasing doses of dabrafenib. Dose-response curves of cell viability according to the sensitivity to dabrafenib is showed for cells knockdown for pre-miR-181a/b (181KD) cluster and controls bearing empty vector (EV, pcDNA3.1-GFP). Data are represented as mean +/− SD, n = 3. (**D**,**E**) Colony formation assays were performed to determine the colony formation ability of A375, A375-BIR and A375-181KD cells transfected with miR-181a and -181b mimics or the appropriate controls. Representative images and quantification of visible colonies have been presented. The colony number in the DMSO group was set as 100%. All the experiments were performed in triplicate and the relative colony formation rates are shown as the mean +/− SD. *** *p* < 0.001; **** *p* < 0.0001; ns, not significative. (**F**,**G**) Effect of forced expression or inhibition of miRNA-181a and -181b on CASP-9 activation in A375, A375-BIR and A375-181KD cells. The determination of CASP-9 activity was carried out by using Caspase-Glo^®^ 9 assay. Data are expressed in fold change relative to vehicle control +/− SD of three independent assays with 3 replicates each one. *** *p* < 0.001; ** *p* = 0.001 and * *p* < 0.01 versus vehicle control; ns, not significant.

Resistant (A375-BIR, M14-BIR) and parental (A375, M14) cell lines were transfected with mimic miR-181a/b and treated with 200 nM dabrafenib. The overexpression of miR-181a/b significantly inhibited melanoma cell growth and enhanced the sensitivity of both sensitive (A375, M14) and resistant (A375-BIR, M14-BIR) cells to dabrafenib (Figure 3A,B, Supplementary Materials Figure S1F,G). Notably, the viability of resistant cells (A375-BIR, M14-BIR) transfected with mimic miR-181a/b and treated with dabrafenib was reduced by more than 50% with respect to treated controls (Figure 3A,B, Supplementary Materials Figure S1F,G).

At the same time, we tested the effect of miR-181a/b silencing in sensitive and resistant melanoma cells. Results showed in Figure 3A,B and Supplementary Materials Figure S1F,G revealed that depletion of miRNA-181a/b in A375 and M14 cells markedly enhanced the proliferation even in the presence of dabrafenib, thus demonstrating a promoting effect on resistance. On the other hand, in both resistant A375-BIR and M14-BIR cells, no significant effect on proliferation was encountered after treatment with dabrafenib compared to controls (Figure 3A,B). The lack of a significant effect after inducing forced down-regulation could be explained as the levels of miR-181a/b expression in resistant cells are very low (Figure 1C–E).

In order to assess the functional role of miR-181a/b in modulating the response to dabrafenib, we established stable A375 and A375-BIR cell lines with permanent knockdown of the pre-miRNA-181a/b gene cluster. To this end, we generated a miR-181a/b “sponge.” MiRNA sponges are tandemly repeated miRNA antisense sequences that can sequester miRNAs from their endogenous targets and are valuable tools to achieve long-term miRNA loss-of-function [25,26]. Sponge design has been carried out as previously reported [25,27]. Briefly, we designed 5 miR-binding sites (MBS) antisense to miR-181a and 5 MBS antisense to miR-181b with a central mismatch at position 9–12 of the corresponding mature miRNA sequences. The two MBS are separated by a 4 nt sequence. Sponge oligonucleotide duplexes were cloned into the 3′ UTR of a GFP in the pcDNA3.1-GFP vector.

A375 and A375-BIR cells were transfected with expressing plasmids containing miRNA-181a/b sponge (A375-miR181-KD; A375-BIR-miR181-KD). PcDNA3.1-GFP vector was used as a negative control (EV). Knock-down efficiency was validated using RT-PCR (Supplementary Materials Figure S1D).

We next performed a dose-response proliferation assay by treating A375- and A375-BIR-miR181KD cells with increasing concentrations of dabrafenib (Figure 3C). As expected, in A375-EV cells, dabrafenib induced a dose-dependent inhibition of proliferation, reporting a GI_50_ of 10 nM, while A375-181KD cells displayed a further increase of proliferation at all drug concentrations tested (GI_50_ > 5 µM), thus strongly suggesting that silencing of both miRNAs induces resistance in melanoma cells (Figure 3C). In agreement with our previous findings, proliferation of A375-BIR-EV cells, expressing the empty vector, was not affected by dabrafenib concentrations up to 10 µM (Figure 3C). Interestingly, we found that silencing of the miRNA-181a/b, significantly increased the proliferation also in A375-BIR-181KD cells even at concentrations higher than 10 µM (Figure 3C).

To confirm these data, we carried out in vitro colony formation assays (Figure 3D,E). The assay was performed by transfecting A375, A375-BIR and A375-181KD cells with miR-181a, 181-b and control mimics, followed by treatment with vehicle control (Veh. CTR) and dabrafenib 200 nM. Representative pictures and their quantification confirmed that miR-181a/b were able to strongly affect colony formation in melanoma A375 and A375-BIR cells (Figure 3D,E). Moreover, forced expression of miR-181a or -181b followed by dabrafenib treatment significantly decreased the number of colonies in both sensitive (A375) and resistant (A375-BIR) melanoma cell lines (Figure 3D,E), thus demonstrating that replacement of miR-181a/b may sensitize melanoma cells to dabrafenib treatment. On the contrary, silencing of miRNA-181a/b significantly induced resistance in A375-181KD cells treated with dabrafenib (Figure 3D,E). In fact, no significant difference in the number of colonies was found in A375-181KD cells treated with dabrafenib compared to those treated with the vehicle CTR. We also tested whether re-expression of miR-181a/b may rescue the resistance of A375-181KD cells to dabrafenib. As showed in Figure 3D,E the re-expression of miR-181a/b followed by dabrafenib treatment markedly decreased the formation of colonies, thus confirming that miR-181a/b replacement may sensitize melanoma resistant cells to dabrafenib.

We further investigated whether the modulation of sensitivity/resistance against dabrafenib operated by miR-181a/b may be correlated to alteration of the apoptotic pathway. A biochemical CASP-9 assay was performed in A375 and A375-BIR cell lines in which miR-181a/b were overexpressed or silenced by transient transfections. As showed in Figure 3F, in A375 cells (dabrafenib sensitive), the forced expression of miR-181a/b markedly increased CASP-9 activity with respect to untransfected cells (Figure 3F). As expected, the pro-apoptotic effect is more pronounced in A375 cells treated with 200 nM dabrafenib for 48 h (Figure 3F). On the contrary, dabrafenib treatment was not able to produce a significant increase of apoptosis in miR-181a/b depleted A375 cells (Figure 3F). We also analyzed whether re-expression of miR-181a/b may induce apoptosis in dabrafenib resistant cells thus sensitizing to the treatment. Our results showed that forced expression of miR-181a/b markedly activated apoptosis in A375-BIR cells treated with dabrafenib, compared with untransfected controls (Figure 3G). We further demonstrated that re-expression of miR-181a/b in A375-181KD cells was able to rescue sensitivity to dabrafenib by inducing apoptosis. Indeed, in A375-181KD cells, dabrafenib treatment failed to induce apoptosis, thus clearly suggesting that depletion of miR-181a/b caused resistance to BRAF inhibitors but more interestingly, the replacement of both miRNAs caused a significant induction of apoptosis in combination with dabrafenib treatment (Supplementary Materials Figure S1E).

Taken together the results described above demonstrated that replacement of 181a/b may reverse the resistance of melanoma cells to dabrafenib, by inducing apoptosis. On the other hand, knockdown of the miR-181a/b cluster gene markedly increased the resistance to dabrafenib, further supporting the functional contribution of both miRNAs to regulating mechanisms of resistance.

### 2.4. Increased Expression of miR-181a/b in Melanoma and Its Relationship with Patient Survival

We next asked whether the miR-181a/b down-regulation observed in dabrafenib-resistant melanoma cells also occurrs in melanoma patients. To address this question, we isolated total RNA from FFPE (Formalin-Fixed Paraffin-Embedded) tumor samples before treatment with kinase inhibitors or from metastatic lesions after relapse in patients who developed resistance to BRAF inhibitors.

The study was conducted on a total of sixteen patients. Among the patients, eight were subjected to dabrafenib or vemurafenib monotherapy, eight were treated with a combination of BRAF inhibitors (dabrafenib, vemurafenib or encorafenib) plus MEK inhibitors (trametinib, binimetinib, cobimetinib) (Table 2). Seven patients had PD (progressive disease) as best response at last tumor assessment, seven had PR (partial response), one had CR (complete response) and one had SD (stable disease) (Table 2). Three patients were subjected to chemotherapy (dacarbazine) or radiotherapy before the targeted therapy. All sample were positive for BRAF mutation. Patients experiencing PR or CR constituted the group of “responders,” whereas patients with PD and SD as best response were included in the group of “resistant.” For the “resistant” group, RNA has been isolated from samples collected before the start of the therapy (T0) and at disease progression (TP).

**Figure 4:**
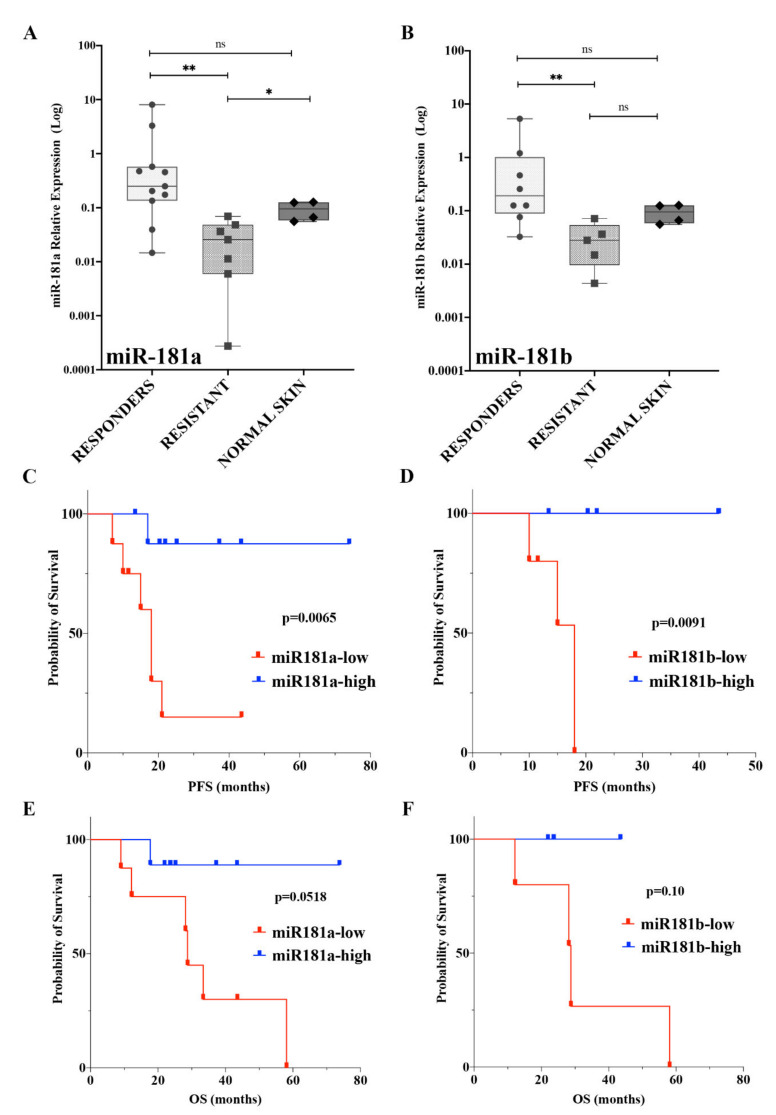
(**A**,**B**) Box-and-whisker diagrams of miRNA-181a (**A**) and -181b (**B**) levels in melanoma patients grouped according to best response to the treatment and in normal skin sample. The horizontal bar within each box indicates the median. Data were analysed by non-parametric Wilcoxon matched-pairs signed-rank test. ** *p* < 0.001; * *p* < 0.01; ns = not significant. (**C,D**) Relationship between the expression of miRNA-181a (**C**) and -181b (**D**) in melanoma tissues and the prognosis of patients. The PFS (progression free survival) rate of the high miRNA-181a (**B**) and -181b (**C**) expression group was higher than that of the low microRNAsexpression group (*p* = 0.0065; *p* = 0.0091). (**E,F**) Kaplan-Meier Log-rank survival analysis for overall survival (OS) of melanoma patients according to miR-181a (**E**) and -181b (**F)** expression (*p* = 0.0518; *p* = 0.1036).

According to data obtained in sensitive and resistant cell lines, the expression profile of mature miR-181a/b in the cohort of patients described above showed significant down-regulation in the “resistant” group (Figure 4A,B). For both miRNAs, the “responder” tumors show high expression levels, while on the contrary, no significant differences can be observed between “responders” and normal skin samples, thus suggesting that high levels of expression may be linked to a positive outcome in clinical practice (Figure 4A,B). A comparison of miR-181a/b expression profiles between the resistant group at T0 and TP was also performed, revealing no significant differences, while a marked down-regulation of both miRNAs was encountered in the comparison of resistant groups and normal skin samples (Supplementary Materials Figure S2A,B), thus indicating that pathways controlling the establishment of resistance in melanoma may negatively regulate the expression of both miRNAs.

Because we have shown above that miR-181a/b expression is associated with the response of tumors to targeted therapy, we postulated a correlation between these miRNAs and the prognosis of melanoma. To test our hypothesis, we assessed whether the expression levels of miR-181a/b are able to predict patient survival, represented by Kaplan–Meier plots. Samples are split in two groups: those with high or low miRNA expression levels. As shown in Figure 4C,D, patients with high levels of expression of miR-181a (n = 9) and -181b (n = 5) were found to have a better survival probability in terms of PFS (progression free survival) than tumors expressing low levels of miR-181a (n = 8) and -181b (n = 5) (Figure 4C,D; *p* = 0.0065; *p* = 0.0091). Among the patients with low levels of miR-181a/b, the median PFS was 18 months, while the median survival could not be estimated due to the limited size of the group and death events (number of death events = 1) (hazard ratio-HR, 0.09868; 95% CI, 0.0213 to 0.4570 comparing the high miR-181a-group versus the low-group; HR, 0.05915; 95% CI, 0.007062 to 0.4955 comparing the high miR-181b-group versus the low-group).

Noticeable improvements were found in high miR-181a/b-group where high expression levels of both miRNAs associated with increased OS (Overall Survival), although the statistical power was limited due to the limited cohort sizes (*p* = 0.0518; *p* = 0.10; Figure 4E,F).

In line with the hypothesis that miR-181a/b may act as tumor suppressors in melanoma, the findings described above demonstrated that expression levels of miR-181a/b may be associated with a positive outcome in melanoma patients treated with the targeted therapy, thus strongly suggesting that miR-181s may be used as predictive biomarkers in the treatment of melanoma.

### 2.5. miR-181a/b Modulate Sensitivity and Resistance to BRAF-Inhibitors by Targeting Key Signalling Networks in Melanoma

To understand the molecular mechanisms adopted by miR-181a/b to regulate the sensitivity and resistance to target therapies in melanoma, we performed a transcriptome profiling of melanoma BRAF-inhibitor sensitive and resistant cell lines. An integrative pipeline correlating miRNAseq and RNAseq data was also established. The analysis has been conducted by using A375 (sensitive) and A375-BIR (resistant) cells, which respectively express high and low levels of miRNA-181a/b as previously assessed by real-time PCR (Figure 1C).

**Figure 5:**
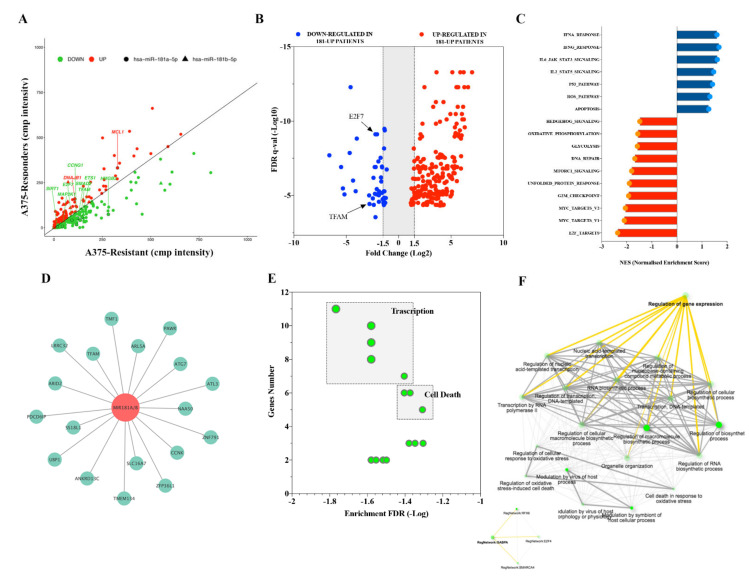
(**A**) Scatter plot of the RNA-Seq expression data showing differentially expressed miR-181a and -181b-target genes (DEG) between A375 (responders) and A375-BIR (resistant) cell lines. Dots represent DEGs that are: significantly overexpressed (red) and down-regulated (green). The most critical upregulated and downregulated DEGs targets are indicated. (**B**) Volcano plot showing the DEGs up (red dots) and down-regulated (blue dots) in patients expressing high levels of miR-181a and -181b (responders group). FDR: False discovery rate. (**C**) Plot of functional gene set enrichment analysis (GSEA) indicating hallmark gene sets significantly modulated in melanoma patients expressing high levels of miR-181a and -181b (responders group). (**D**) Network of miRNA-181a- and -181b-negatively regulated predicted target genes in melanoma patients (responders group) visualized by Cytoscape. Green dots represent predicted target genes and the red dot represents hub miRNA. (**E,F**) GO term enrichment analysis of miRNA-181a- and -181b-negatively regulated predicted target genes in melanoma patients (responders group). (**E**) Scatter plot of enriched GO pathways according to numbers of genes and FDR. (**F**) GO network analysis plot shows the relationship between enriched pathways. Darker green nodes are more significantly enriched gene sets. Bigger nodes represent larger gene sets. Most critical networks are highlighted in yellow.

Differential expression analysis between A375 and A375-BIR cells identified 434 differentially expressed genes (DE) (FDR < 0.05). DE genes were further screened using a common miRNA target prediction engine (TargetScan), thus identifying 222 DE target genes potentially regulated by miR-181a/b expression (Supplementary Materials Table S1; Figure 5A). Among down-regulated genes, the analysis showed several experimentally validated target genes, such as *E2F7* [28], *MAP2K1* (also known as *MEK1*) [29], *CCNG1* [24], *HMGB2* [30], *SIRT1* [22], belonging to key signaling pathways frequently associated to melanoma (Supplementary Materials Table S1; Figure 5A). We also identified 90 DE target genes up-regulated in A375 cells with miR-181a/b high expression levels. Belonging to this group, several molecular players of apoptosis, such as *DNAJB1* and *MCL1* were found (Supplementary Materials Table S1; Figure 5A). Moreover, GO functional annotation analysis identified transcriptional regulation, metabolic process and apoptosis as top ranked gene sets (Supplementary Materials Figure S3A–C). Belonging to transcription factor gene set, the presence of the mitochondrial transcription factor A *TFAM* represents a predicted target of particular interest (Figure 5A). In fact, a recent paper demonstrated that *TFAM* acts as an oncogene by transcriptionally regulating metabolic and mitochondrial gene signatures in melanoma [31].

In order to investigate the role and clinical significance of miR-181a/b we analyzed the expression profile of miR-181a/b and the related targetome in melanoma clinical sample. The expression profile of miR-181a/b in 17 melanoma samples has been retrieved by using real-time expression data as described in the previous paragraph (Figure 4A,B). To avoid bias in this analysis, we selected melanoma samples harboring a mutation in the V600 BRAF codon (n = 17), as all the melanoma cell lines in our previous analysis also carried this mutation. Similar to the analysis described in melanoma cells, we divided the tumors according to the levels of expression of miR-181a/b, applying a cut-off median to select samples with low (miR-181a/b down, n = 9) and high (miR-181a/b up, n = 8) miR-181a/b expression (Figure 4A,B). As described in Figure 4, the expression of miR-181a/b is effectively decreased in “resistant” melanoma, if compared with the “responders” group which showed significantly increased levels of expression.

The transcriptomic profile of the melanoma samples led us to identify 274 DE between responders (miR-181a/b high) and resistant (miR-181a/b low) groups (Figure 5B). Enrichment pathway analysis with the DE revealed key pathways that could be regulated by both miR-181a and miR-181b. By using the MSigDB (Molecular Signature Database), we identified in the ‘responders’ group (miR-181a/b high), several enriched hallmark gene sets including Apoptosis, P53 and ROS, IFNγ and IFNα response, IL6/JAK/STAT3, IL2/STAT5 (Figure 5C, Table 3). Representative inhibited processes included cell cycle regulators such as those involved in the G2M checkpoint, E2F and MYC targets. Metabolism-related processes such as Oxidative Phosphorylation and Glycolysis gene sets were also found to be down-regulated (Figure 5C, Table 3). Interestingly, two signaling pathways, commonly associated with drug-resistance, the DNA repair and Unfolded Protein Response pathways were negatively enriched. Importantly, there were significant inverse correlations between miR-181a/b expression and melanoma-related oncogenic pathways as mTOR and Hedgehog (Figure 5C; Table 3).

Furthermore, we analyzed the expression of miR-181a/b-predicted target genes in the melanoma samples mentioned before and found a negative relationship between expression levels of miR-181a/b and eighteen predicted target genes (Table 4; Figure 5D; *p* < 0.05). Investigating negative-regulated target genes, we found eight genes (*ANKRD13C*, *ARL5A*, *ATL3*, *PAWR*, *PDCD6IP*, *SLC16A7*, *TFAM*, *UBP1*) presenting a significant inverse correlation with miR-181a/b expression also in melanoma A375 sensitive cells (Supplementary Materials Table S1; Figure 5D). To get insights into the functional role of miR-181a/b in drug-resistance in melanoma, the subset of 18 selected target mRNAs mentioned above was tested for overrepresentation in databases representing biological processes. Our analysis indicated that the negatively regulated targets were significantly enriched in 31 pathways from GO (gene Ontology) (Supplementary Materials Table S3). Importantly, among the top ranked pathways, we identified several processes of significance for regulation of transcription, regulation of apoptotic processes and mitochondrial functions (FDR < 0.05; Figure 5E-F; Supplementary Materials Table S2). Indeed, among the enriched gene sets we also found the regulation of cellular response to oxidative stress pathway (Figure 5E,F; Supplementary Materials Table S2). Collectively our results suggest that miR-181a/b levels may target a transcription factor regulatory network, thus specifically regulating the expression of key components of molecular pathways frequently associated to the development of drug-resistance in melanoma.

### 2.6. MiR-181a/b Regulate Melanoma Resistance by Targeting TFAM

**Figure 6:**
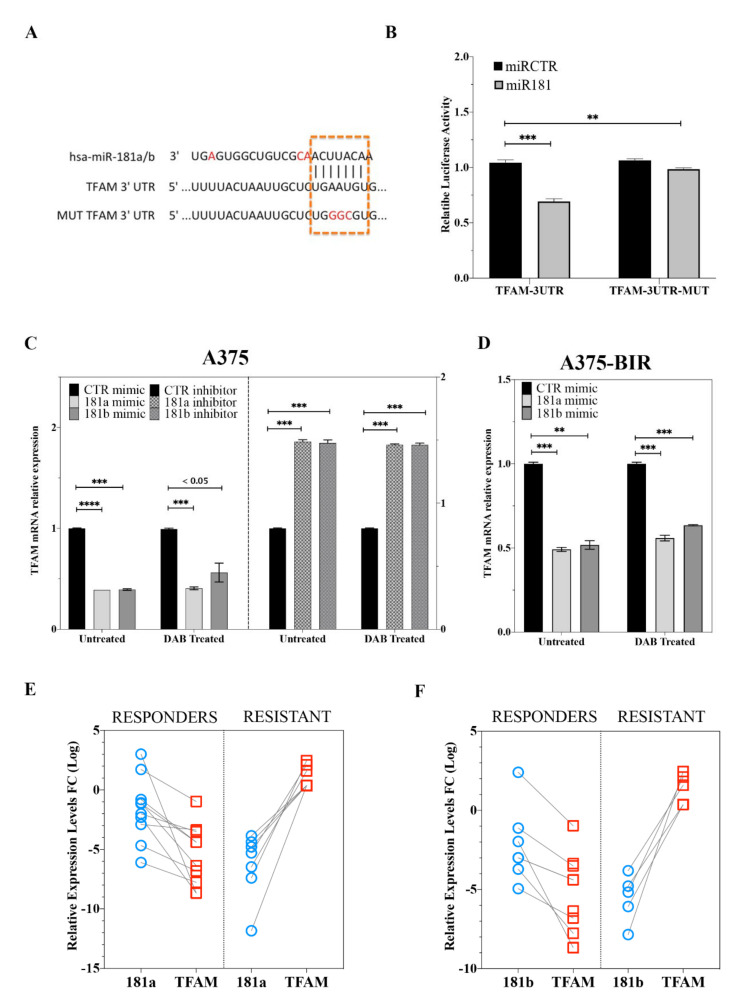
(**A**) TFAM 3′UTR contains miR-181a and -181b binding sites. Red-labelled nucleotide indicate the differences between the sequences of miR-181a and -181b. (**B**) The dual luciferase reporter assay was performed with HeLa cells as described in Materials and Methods. Briefly, HeLa cells were transfected with miR-181a or miR mimic negative control together with the plasmid encoding for the Firefly luciferase (FFL) carrying part of the TFAM 3′-UTR in its wild type form (TFAM-3UTR) or TFAM 3ʹ-UTR with the mutagenized form of the miR-181a and -181b binding site (TFAM-3UTR-MUT). FFL activities were internally normalized to Renilla luciferase activities yielding relative light units (RLU). Histograms show the mean +/− SE from 3 to 6 independent experiments upon normalization to the miR mimic control. ** *p* < 0.01; *** *p* < 0.001 in un-paired t-test. (**C**,**D**) TFAM mRNA expression analysis in A375 (**C**) and A375-BIR (**D**) cells transfected with miR-181a, -181b mimics or their inhibitors or relative controls. Histograms show the mean +/− SE from 3 independent experiments upon normalization to the miR mimic control. ** *p* < 0.01; *** *p* < 0.001; **** *p* < 0.0001 in un-paired t-test. (**E**,**F**) Treatment resistant and responder melanomas display miR-181a/b/*TFAM* axis deregulation. Lower miR-181a (**E**) and -181b (**F**) and higher TFAM expression levels in patients resistant during therapy compared to tumors from the responder group. *p* < 0.001 by Mann-Whitney U test.

As stated before, it has been shown that *TFAM* acts as an oncogene in melanoma regulating metabolic and mitochondrial genes [31]. We thus decided to experimentally validate TFAM as direct miR-181a/ target. Potential targets of miR-181a/b were predicted using three bioinformatic databases (TargetScan, miRanda and PicTar). *TFAM* was chosen as a target gene of miR-181a/b, based on putative target sequences of *TFAM* localized in the 3′UTR (Figure 6A). To verify whether *TFAM* is a direct target of miR-181a/b, a human *TFAM* 3′UTR fragment containing the binding site of the two miRNAs (Figure 6A) or the same construct carrying a mutant binding site (negative control) were cloned into the pGL3 vector and the miR-181a mimic or miR-CTRL were co-transfected into HeLa cells and cultured for 48 h. Then luciferase activities were measured by a luminescent assay. As expected overexpression of miR-181a suppressed the luciferase activity of wild-type *TFAM* 3′UTR but the activity of the mutant-type *TFAM* 3′UTR was not changed (Figure 6B), indicating *TFAM* as direct miR-181a/b target. Then, qRT-PCR analysis confirmed that overexpression of miR-181a/b markedly inhibited *TFAM* expression on mRNA level in both sensitive (Figure 6C) and resistant (Figure 6D) A375 melanoma cells. Moreover, depletion of miRNA-181a/b in A375 cells resulted in significantly increased expression of *TFAM*. Real time expression analysis also demonstrated that the negative modulation of *TFAM* expression was not affected by dabrafenib treatment (Figure 6C,D). These results indicate that miR-181a/b binds directly to TFAM and inhibits its expression (Figure 6C,D).

Next, we investigated the clinical significance of *TFAM* targeting by miR-181a/b in melanoma using the patient cohort described in Table 2. *TFAM* mRNA expression levels were significantly increased in “resistant” patients compared to the “responders” group (Figure 6E,F). The *TFAM* mRNA levels were significantly correlated with miR-181a/b levels (Figure 6E,F). The negative correlation between both miRNAs and *TFAM* was highly significant in the “responders” subtype (Figure 6E,F). On the contrary, in the “resistant’ group with low miR-181a/b expression, TFAM mRNA expression was significantly increased, thus demonstrating an inverse correlation.

Our results strongly support a central role for the miR-181a/b-*TFAM* axis in modulating key components of tumor growth with target therapy resistance both in vitro and in vivo melanoma models.

## 3. Discussion

The development of therapeutic strategies targeting BRAF and MEK and the introduction of immunotherapy represented a big challenge in the treatment of melanoma. Targeted agents and immunotherapies, administered as single agents or their combination, markedly improved the outcome, increasing progression-free (PFS) and overall survival (OS) of melanoma patients [4]. However, in the majority of cases, the clinical benefit of these agents is severely limited by the occurrence of intrinsic, adaptive and acquired resistance mechanisms [32]. Melanoma exhibits dynamic plasticity regulated by genetic, epigenetic and transcriptional changes; this confers a high adaptability to a wide range of anti-cancer agents, including target- and immuno-therapies [33]. Interestingly, this dynamic phenotype involves fundamental cellular processes such as cell proliferation, differentiation and metabolic rewiring, thus allowing melanoma cells to robustly resist all current therapies. Emerging evidence has demonstrated that miRNAs might be responsible for the fine-tuning of the cellular processes described above which regulate resistance in melanoma [8,9].

In this study, we further explored the role of miRNAs in melanoma resistance to BRAF inhibitors (BRAFi). Comparing miRNA expression profiles of a BRAFi-sensitive and dabrafenib-resistant cell line, we identified 65 miRNAs whose expression was significantly altered (Figure 1, Table 1). Belonging to differentially expressed miRNAs we identified several microRNAs, including miR-126, miR-146a; miR-424, miR-503, miR-509, miR-224, already reported to be involved in the onset and/or progression and resistance of melanoma [17,18,19,20,21], thus confirming the validity of our approach (Table 1). In order to identify novel miRNAs potentially involved in melanoma resistance, we focused our attention on miRNA-181a and -181b, whose involvement in cancer and drug-resistance has been demonstrated in a widely range of tumors [13,15,22,23,24]. Indeed, altered expression of miR-181a/b has been discovered in several kinds of cancers. Moreover, members of the miR-181 family have been investigated as prognostic biomarkers in Non-Small-Cell Lung Carcinoma (NSCLC) [13], Colorectal cancer (CRC) [13], Bile Duct Cancer (BDC) [12] and the up-regulation of miR-181 family members was recently shown to be associated with chemotherapy response in patients affected by gastric cancer. Different studies have shown that aberrantly high miR-181a expression can regulate cell cycle, apoptosis, invasion, proliferation and metastasis in several kinds of cancer [13,15,22,23,24]. The expression of miR-181a in melanocytes and melanoma cells has been investigated by Bosserhoff et al. [34] who reported the down-regulation of miR-181a during the progression of melanoma thus hypothesizing a tumor-suppressing role for this miRNA. In this paper, we first carried out functional studies in melanoma cell lines to investigate the molecular role of miR-181a/b in melanoma, also raising evidence of their involvement in resistance to BRAF inhibitors-resistance in vitro and in clinical samples. Human miR-181a and -181b are transcribed in clusters at two genomic loci on chromosomes 1 and 9 and share the same seed region modulating a similar set of target mRNAs [13]. These miRNAs are emerging as onco-suppressors in multiple cancer types, including NSCLC, CRC, Brain Cancers and Hematological Cancers [13]. In line with these reports, we found that both miRNAs are significantly down-regulated in resistant melanoma patients and cell lines. Furthermore, miR-181a/b replacement in melanoma cells resistant to BRAFi, marked suppressed cell viability and colony formation, by inducing cell death and activating apoptotic machinery (Figure 2 and Figure 3). The forced expression of both miRNAs increased sensitivity to dabrafenib, strongly suggesting a potential use as therapeutic strategy (Figure 2 and Figure 3). In light of the discussed aspects, we are proposing a novel type of combined therapy by merging the effects of miRNA replacement in combination with dabrafenib to obtain a superior inhibitory effect upon cancer cells. Therapeutic strategies using microRNAs as drugs is currently becoming a reality as revealed by several ongoing clinical trials currently investigating the efficacy and safety of miRNA-based therapies [32,33,34]. In particular, the therapeutic strategies developed to replace miRNAs with tumor suppressive functions involve the use of chemically-modified oligonucleotide duplexes mimicking the onco-suppressive function of naturally occurring miRNAs [32,33,34,35,36].

In the current paper, the tumor-suppressive role of miR-181a/b has also explored in clinical sample of melanoma, thus revealing that high levels of these miRNAs were associated with favorable clinical responses in melanoma patients treated with BRAFi or BRAFi plus MEKi (Figure 4 and Figure 5). Despite to be limited by the small cohort of sample, we showed a marked positive correlation between miR-181a/b expression and survival of melanoma patients, thus also suggesting a potential use of both miRNAs as biomarkers for predicting the response to target therapy in melanoma. Several authors proposed the expression level of the miR-181a as a potential biomarker for assessing prognosis and therapeutic response in multiple cancers such as breast cancer, human cervical squamous cell carcinoma, CRC and endometrial cancer [24,37,38,39]. In line with this evidence, our findings strongly suggested the potential use of miRNA-181a and -181b as novel potential biomarkers to predict disease outcome and therapeutic response in the clinical management of melanoma.

Our analysis of the functional role of miR-181a/b also investigated their regulatory network, thus identifying key signaling pathway regulating melanoma progression and resistance. In particular, a network of genes, involved in transcriptional regulation of metabolic processes and cell death, has been found to be negatively enriched in melanomas with high expression levels of miR-181a/b (Figure 5; Supplementary Materials Figure S3). We also showed that the miR-181a/b functions are determined by the direct binding to their target Mitochondrial transcription factor A (*TFAM*). *TFAM* is a well-known transcription factor driving the transcription and replication of mtDNA, the regulation of mtDNA copies and the maintenance/repair of mitochondrial genes [40]. Moreover, *TFAM* also has an important role in maintaining the functional integrity of mitochondrial respiratory chain and the balance between anti-oxidation and oxidation [40]. A recent work of Araujo et al., reported that *TFAM* regulates the expression of key genes involved in cell metabolism, mitochondrial DNA (mtDNA) instability and metabolic changes that contribute to tumorigenesis [31]. Our results provide evidence of a negative correlation between miR-181a/b expression and their target *TFAM* in patients with a positive outcome and responding to targeted therapies (Figure 6), thus strongly suggesting that miR-181a/b exert their tumor-suppressive action by targeting *TFAM* and thus silencing its pro-tumorigenic functions. Importantly, a seminal work of Herlyn M. et al. showed that TFAM is able to induce resistance to BRAF- and MAPK- inhibitors by regulating the mitochondrial biogenesis in melanoma [41]. The same authors also provided evidence that targeting *TFAM* may be a promising therapeutic approach to overcome resistance in melanoma. Our group has recently demonstrated that miR-181a/b control target genes implicated in mitochondrial biogenesis, clearance, functionality and antioxidant response, uncovering a fundamental role of miR-181a/b in the control of autophagy/mitophagy, mitochondrial cell death and mitochondrial functionality [42]. Notably mitochondria are emerging as important determinants of several aspects of cancer development and progression, including metabolic reprogramming, acquisition of metastatic capability and response to therapeutic agents [8,17,19,43,44]. These observations clearly suggest that miR-181a and -181b might regulate several key-mitochondria related fundamental functions, notably associated to a tumor suppressive role and to a positive response to therapeutic agents in melanoma.

In summary, our results demonstrated for the first time that miR-181a/b expression was decreased in melanoma cells resistant to BRAFi and their low expression contributed to BRAFi resistance. Furthermore, by targeting *TFAM*, miR-181a/b replacement has a suppressive role in melanoma growth. Moreover, high expression levels of miRNA-181a and -181b were associated with an increased OS and PFS, while the low expression to a poor outcome and marked resistance to target therapies, thus showing for the first time that both miRNAs may represent a promising biomarker for predicting patients’ response to BRAFi or BRAFi + MEKi and for monitoring the onset of resistance in melanoma. Furthermore, we provided evidences that miR-181a and -181b may represent new and potentially powerful candidates for therapeutic intervention to overcome resistance in refractory melanomas.

## 4. Materials and Methods

### 4.1. Human Samples

Total RNA was extracted from the FFPE (Formalin-Fixed-Paraffinn-Embedded) samples from 17 matched tumors from patients before and after the development of resistance to BRAF inhibitors as single agent or in combination with MAPK inhibitors (see Table 2). MiRNAs were extracted from FFPE melanoma tissues before the beginning of therapy and at disease progression through Maxwell^®^ RSC RNA FFPE Kit (Promega, Madison, WI, USA) and processed with Maxwell^®^ RSC Instruments (Promega, Madison, WI, USA) according to the manufacturer’s instructions. Real-time PCR was assayed as described above. The use of human samples was approved by the Ethics Committee of Istituto Nazionale Tumori—IRCCS—Fondazione "G. Pascale", Naples, Italy (date of registration 18/01/2018; project identification code n° 33/17 oss, 2018. Written informed consent was obtained from all participants.

### 4.2. Cell Culture and Transfections

A375, M14 cells were obtained from American Type Culture Collection (ATCC) (Manassas, VA, USA) and cultured RPMI 1640 (Invitrogen, Karlsruhe, Germany) with L-Glutamine, 10% fetal bovine serum (FBS; Invitrogen, Carlsbad, CA, USA), 100 U/mL penicillin and 50 µg of streptomycin, at 37 °C in humidified 5% CO_2_ atmosphere. HeLa cells (No. CCL 185; American Type Culture Collection, Rockville, MD, USA) were grown in Dulbecco’s modified Eagle’s medium supplemented with 10% fetal bovine serum (FBS) and 2 mM L-glutamine (GIBCO BRL, Life Technologies) at 37°C and 5% CO2.

Dabrafenib-resistant melanoma cells (A375- and M14-BIR) were selected by growing melanoma cell lines in medium containing increased concentrations of dabrafenib for 4 weeks. A375 and M14 melanoma cell lines resistant to 1 µM dabrafenib were selected. Dabrafenib was purchased from Selleck (Munich, Germany). 

Authentication of cell lines was done by Eurofins (Milan, Italy) and confirmed by online STR-matching analysis (www.dsmz.de/fp/cgi-bin/str.html).

MiR-181a and -181b mimics, inhibitors and negative controls were purchased from Dharmacon. Transfection with 40 nM mimics or inhibitors and/or negative control mimics was performed with Lipofectamine 2000 reagent (Invitrogen, Karlsruhe, Germany). MiR-181a/b sponge was synthetized by Genewiz and cloned into the pcDNA3.1-GFP. The sequence is covered by the patent application “miR-181 inhibitors and uses thereof” (WO2019202162A1).

### 4.3. RNA Extraction and Quantitative Reverse Transcription PCR

Total RNA was extracted from the cell samples using Trizol reagent (Invitrogen, California, CA, USA) [45] or in alternative Maxwell RSC simplyRNA Cells (Glomax Discover System, Promega, Madison, WI, USA) and processed with Maxwell RSC Instrument (Glomax Discover System, Promega, Madison, WI, USA) following manufacturer’s instructions. Single-stranded complementary DNA (cDNA) was generated using Quantitect Reverse Transcription kit (Qiagen, Germantown, MD, USA). Quantitative RT-PCR was performed in LightCycler 96 (Roche, Penzberg, Germany) using LightCycler FastStart DNA Master SYBR Green I (Roche, Penzberg, Germany) and each validated primer. Validated qRT-PCR primers of TFAM, GAPDH and HPRT1 were from Eurofins (Milan, Italy).

Total RNA from FFPE tissues was isolated using Maxwell RSC RNA FFPE kit (Promega, Madison, WI, USA) and processed with Maxwell RSC Instrument (Promega, Madison, WI, USA).

For miRNA quantification, small RNA was isolated with miRNA Tissue Kit (Promega) according to the manufacturer’s instructions. Quantitative RT-PCR for miRNA was performed using a TaqMan MicroRNA assay kit (Applied Biosystems, Foster City, CA, USA) and specific primer sets for U6 snRNA (Assay ID: 001973), mature miR-181a (Assay ID: 000480), miR-181b (Assay ID: 001098), miR-224 (Assay ID: 121210-mat) (Applied Biosystems, Foster City, CA, USA) according to the manufacturer’s instructions [46]. We used HPRT1 and U6 snRNA as internal normalizers for mRNA and miRNA, respectively.

### 4.4. Proliferation Assay

MTS assays were performed using tetrazolium-based CellTiter 96 AQueous One Solution Cell Proliferation assay (Promega, Madison, WI, USA). Cells were seeded in 96-well plates at 8,000 cells per well for overnight incubation. Following adhesion of cells to the well, cells were treated with the experimental treatments indicated. Control groups were exposed to the same concentration of DMSO (Dimethyl sulfoxide; Sigma-Merck KGaA, Darmstadt, Germany). At the designated time-points, plates were read at the absorbance of 492 nm on a microplate reader (Glomax Discover System, Promega, Madison, WI, USA). Relative cell viability of an individual sample was calculated by normalizing their absorbance to that of the corresponding control. All experiments were done in triplicate. The GI50 was determined from the regression of a plot of the logarithm of the concentration versus percent inhibition by Graph Pad Prism (version 8) using the Dose-Response One-Site Model.

### 4.5. Colony Formation Assay

The cells were seeded onto 24-well plates (200 cells/well) and treated with indicated compounds or vehicle controla (Dimethyl sulfoxide, DMSO; Sigma-Merck KGaA, Darmstadt, Germany) for 7-10 days. After washing and fixation, the cells were stained with 0.5% Crystal Violet (Bio Basic Inc., Markham, Canada) in 25% methanol for 10 min. Cell colonies were then photographed and counted.

### 4.6. Caspase Activity Assay

Following treatments cells were subjected to Caspase 9 activities measurement with Caspase-Glo assay kit (Promega, Madison, WI, USA). Briefly, the plates containing cells were removed from the incubator and allowed to equilibrate to room temperature for 30 min. 50 µL of Caspase-Glo reagent was added to each well, the content of well was gently mixed with a plate shaker at 300–500 rpm for 30 s. The plate was then incubated at room temperature for 2 h. The luminescence of each sample was measured in a plate-reading luminometer (Glomax Discover System, Promega, Madison, WI, USA) with parameters of 1 min lag time and 0.5 s/well read time. The experiments were performed in triplicate and repeated on two separately-initiated cultures.

### 4.7. RNAseq and Small-RNAseq Library Preparation and Deep Sequencing

For RNA-seq analysis, libraries were prepared according to the manufacturer’s instructions (QuantSeq 3’ mRNA-Seq Library Prep Kit FWD for Illumina, Lexogen GmbH, Wien, Austria) starting from 250 ng of total RNA. Quality control of library templates was performed using a High Sensitivity D1000 Screen Tape (Agilent Technologies, Santa Clara, CA, USA) on a TapeStation 4200 (Agilent Technologies, Santa Clara, CA, USA). The Qubit quantification platform (Qubit 2.0 Fluorometer, Life Technologies/Thermo Fisher Scientific, MA, USA) was used to normalize samples for the library preparation. Using multiplexing, up to 87 samples were combined into a single lane to yield sufficient coverage. The sequencing was carried out in collaboration with the Next Generation Diagnostic (NGD, Pozzuoli, Naples, Italy). Libraries were sequenced by single-end chemistry on an NovaSeq6000 platform (SP 100 cycles; Illumina, Cambridge, UK). Each library was loaded at a concentration of 250 pM, which was previously established as optimal. An average yield of ~4.5 Mb was obtained per sample.

Small RNA-Seq was performed by using a Small RNA-Seq Library Prep Kit (Lexogen, GmbH, Wien, Austria), according to manufacturer protocols. To verify the quantity and quality of RNA extracted before the library construction, the samples were tested using 2100 Bioanalyzer “total RNA pico bioanalyzer kit”(Agilent, Santa Clara, CA, USA) and the concentrations of all RNA solutions were determined using a Qubit 2.0 fluorometer (Life Technologies/Thermo Fisher Scientific, MA, USA). The RNA samples were used to produce cDNA libraries using the Small RNASeq Library Prep kit (Lexogen GmbH, Wien, Austria) according to the user manual. Input RNA was primarily ligated to a 3′ adapter then, after removing excess 3′ adapter by column purification, it was ligated to 5′ adapter and the excess was removed. In the second step the RNA, flanked by 5′ and 3′ adapters, was converted into cDNA during the PCR amplification step through the adjunction of multiplexing indices. These indices are used to distinguish the single samples after the pooling phase. The library product was cleaned-up and concentrated with gel-based purification protocol. This step removes linker-linker artefacts (120 bp) and long library fragments (>200 bp). The presence of linker-linker artefacts in the library may reduce the power of amplification and the consequent sequencing results. A size selection using a 6% Tris-Glycine-SDS Precast Polyacrylamide Gel (Life Technologies/Thermo Fisher Scientific, MA, USA) was used to achieve this goal according to the Library prep instruction manual and using a Gel extraction module compatible with PAGE gel purification (Lexogen GmbH, Wien, Austria). The sequencing step was performed with NGS technologies using Illumina Novaseq 6000 SP kit (100 cycles) produced by Illumina (Illumina, Cambridge, UK).

### 4.8. Computational Analysis of Deep Sequencing Data

A data analysis was performed using the pipeline already established at the Bioinformatics and Statistics Core Facility at TIGEM [47]. Briefly, the reads were trimmed to remove adapter sequences and low-quality ends and reads mapping to contaminating sequences (e.g., ribosomal RNA, phIX control) were filtered out. Reads were aligned and assigned to Human ENSEMBLE transcripts and genes (hg38 reference) by using RSEM version 1.2.25 with standard parameters. The threshold for statistical significance chosen was False Discovery Rate (FDR) < 0.05. The Gene set enrichment analysis (GSEA) was then performed restricting the output to the collection of “hallmark” and “Biocarta” gene sets part of the Molecular Signatures Database (MSigDB v7.0). The threshold for statistical significance chosen in the GSEA was False Discovery Rate (FDR) < 0.25. The expression of differentially induced/suppressed genes (FDR < 0.05) was validated by RT-PCR. The data have been deposited in NCBIs Gene Expression Omnibus (GEO): Superseries GSE165338: RNAseq and Small-RNAseq dataset to comprehensively study the miRNA expression profiling of drug-resistant melanoma patients and cell lines. GSE165334: Transcriptome profile of melanoma cell lines A375 and A375-BIR (BIR-BRAF INHIBITOR RESISTANT) during dabrafenib treatment. GSE165335: SmallRNAome profile of melanoma cell lines A375 and A375-BIR (BIR-BRAF INHIBITOR RESISTANT) during dabrafenib treatment. GSE165337: Transcriptomic profile of melanoma clinical samples treated with BRAF and MEK- inhibitors.

### 4.9. Identification of Differentially Expressed miRNAs and Specific miRNAs

We compared the miRNA expression profiles between the A375 and A375-BIR cells in both control (DMSO) and treatment groups by using the EdgeR package (https://www.bioconductor.org/packages/release/bioc/html/edgeR.html) under software R (version 4.0.2) [48]. MiRNAs that have very low counts across all the libraries were removed prior to perform normalization by trimmed mean of M values (TMM) [48]. The filtering is based on count-per-million (CPM) values and it is necessary to avoid miRNAs that are expressed in larger libraries over those expressed in smaller libraries. We considered miRNAs as differentially expressed between cell lines when FDR is less than 0.05. More precisely, we identified an miRNA as specific to a cell line when it was mainly over-expressed or under-expressed in the treatment group compared with the placebo group following these criteria: log2 fold change >2 or <−2 and FDR < 0.05.

### 4.10. Integrated Analysis of miRNA and mRNA Based on Correlation between mir181a/b and Related Target Gene in Melanoma Patients

Correlation analysis of mir181a/b and predicted target genes based on the mRNA-miRNA interaction network was performed by considering the expression profile in melanoma patients. The network was constructed by considering the negative statistically significantly correlation (Pearson’s correlation) between miRNA and mRNA expression, which included mir181a/b and 18 predicted target mRNAs. To create the interaction network, we converted the read counts into counts-per-million (CPM) values for both mRNA and miRNA data. Cytoscape tool was used to build network of interaction (Cytoscape 3.8.2 version, https://cytoscape.org). Targetscan 7.2 (http://www.targetscan.org/vert_72/) was used to identify predicted targets. The signaling pathway of mRNA targets was analysed using GO Enrichment analysis (https://biit.cs.ut.ee/gprofiler/gost).

### 4.11. Reporter Assay

To prepare the reporter constructs, the 3′UTR of target genes containing the putative miR-181 binding sites were amplified by PCR and HeLa cDNA as a template. The amplified PCR products were cloned the downstream of the luciferase gene in the pGL3-luc vector (Promega, Madison, WI, USA) as schematically depicted in Figure 6 [12]. For generation of the mutant reporters, three nucleotide mutations were introduced into the putative miR-181 binding sites using a QuikChange II XL site-directed mutagenesis kit (Agilent Technologies, Santa Clara, CA, USA). All primers were purchased from Eurofins (Milan, Italy). HeLa cells were cotransfected with reporter plasmid (200 ng), pRL-CMV-Renilla plasmid (10 ng) and 10 nM miRNA in 96-well plates using Lipofectamine 2000 (Invitrogen, California, CA, USA) according to the manufacturer’s instructions. After 48 h of transfection, luciferase activity was measured using a Dual Luciferase Reporter Assay system (Promega, Madison, WI, USA) according to the manufacturer’s instruction. Firefly luciferase activity was normalized to Renilla luciferase activity.

### 4.12. Statistical Analysis

Statistical tests including Student’s *t* test and one-way ANOVA with Bonferroni’s multiple comparison test were performed with Prism GraphPad (GraphPad Software 8.0, La Jolla, CA, USA). Data are presented as mean +/− SD. Results were considered statistically significant if *p* < 0.05.

The calculation of the GI_50_ values were performed with GraphPad Prism (GraphPad Software 8.0, La Jolla California, USA) and followed a nonlinear regression model applied to the sigmoidal dose-response curves of the cell viability data. The values were log-transformed before fitting the model.

### 4.13. Survival Analysis

For survival data, Kaplan–Meier curves were plotted and compared using a log-rank test. All tests were two-sided. To evaluate the discrimination of the miRNA-based prediction signatures in melanoma patients we performed a log-rank test between high- and low-mi181a/b risk groups. We stratified patients into high- and low-risk subtypes with median risk score. Kaplan-Meier curve was used to compare the OS (overall survival) and PFS (progressive free survival) between the high- and low risk groups. All analysis was performed with R software (version 4.0.2) using survival R package. A *p*-value < 0.05 was considered statistically significant.

## Figures and Tables

**Table 1:** Differentially Expressed microRNAs between A375 and A375-BIR melanoma cell lines.

MicroRNA ID	logFC	FDR	Regulation
microRNA 224	−5.863656346	2.89 × 10^−5^	DOWN
microRNA 1270	−5.796946495	5.23 × 10^−5^	DOWN
microRNA 31	−4.893572244	1.68 × 10^−86^	DOWN
microRNA 130a	−4.666019353	6.41 × 10^−7^	DOWN
microRNA 1269a	−4.482898282	1.25 × 10^−21^	DOWN
microRNA 3929	−3.74725201	4.40 × 10^−5^	DOWN
microRNA 33a	−3.475625978	2.69 × 10^−2^	DOWN
microRNA 622	−2.886943306	5.68 × 10^−4^	DOWN
microRNA 30a	−2.703339809	2.05 × 10^−24^	DOWN
microRNA 505	−2.627997044	1.89 × 10^−3^	DOWN
microRNA 196b	−2.528703752	3.84 × 10^−11^	DOWN
microRNA 210	−2.469223892	8.77 × 10^−8^	DOWN
microRNA 182	−2.461507371	2.54 × 10^−38^	DOWN
microRNA let-7c	−2.393014183	1.88 × 10^−31^	DOWN
microRNA 146b	−2.268652948	2.10 × 10^−18^	DOWN
microRNA 15b	−1.995340697	3.87 × 10^−10^	DOWN
microRNA 183	−1.969984406	1.46 × 10^−3^	DOWN
microRNA 222	−1.886429667	3.50 × 10^−28^	DOWN
microRNA 424	−1.683371	4.29 × 10^−5^	DOWN
microRNA 30c-2	−1.67640615	3.28 × 10^−11^	DOWN
microRNA 34a	−1.668911618	4.39 × 10^−6^	DOWN
microRNA 29a	−1.631521943	4.39 × 10^−6^	DOWN
microRNA 30c-1	−1.616623692	6.56 × 10^−9^	DOWN
microRNA 125b-2	−1.434360093	1.65 × 10^−7^	DOWN
microRNA 20a	−1.264913487	2.95 × 10^−2^	DOWN
microRNA 100	−1.24322378	9.16 × 10^−6^	DOWN
microRNA 221	−1.185152679	2.29 × 10^−5^	DOWN
microRNA 27a	−1.177996843	1.30 × 10^−4^	DOWN
microRNA 125b-1	−1.133330102	5.88 × 10^−8^	DOWN
microRNA 24-2	−1.121098474	1.01 × 10^−6^	DOWN
microRNA 769	−1.116258565	3.42 × 10^−4^	DOWN
microRNA 93	−1.087377219	1.17 × 10^−2^	DOWN
microRNA 1307	−1.077056378	9.18 × 10^−5^	DOWN
microRNA 196a-2	−0.782047919	3.08 × 10^−3^	DOWN
microRNA 30e	−0.737216081	1.48 × 10^−2^	DOWN
microRNA let-7a-2	−0.732269399	2.66 × 10^−5^	DOWN
microRNA let-7a-1	−0.729090427	3.26 × 10^−5^	DOWN
microRNA let-7a-3	−0.724904576	3.19 × 10^−5^	DOWN
microRNA let-7b	0.551945479	3.73 × 10^−2^	UP
microRNA 197	0.655730622	1.36 × 10^−2^	UP
microRNA 612	0.797582759	2.93 × 10^−2^	UP
microRNA 663a	0.855598474	3.68 × 10^−4^	UP
microRNA let-7f-1	0.873310731	3.20 × 10^−6^	UP
microRNA let-7d	0.87390186	5.69 × 10^−7^	UP
microRNA let-7f-2	0.904197222	1.14 × 10^−6^	UP
microRNA 30b	0.947032776	1.15 × 10^−3^	UP
microRNA 1180	1.099377126	1.46 × 10^−5^	UP
microRNA 140	1.253304012	5.15 × 10^−5^	UP
microRNA 345	1.308512546	1.85 × 10^−2^	UP
microRNA 98	1.44563401	9.26 × 10^−8^	UP
microRNA 199b	1.624916736	9.50 × 10^−4^	UP
microRNA 199a-2	1.945384932	1.24 × 10^−4^	UP
microRNA 199a-1	1.978424668	8.68 × 10^−5^	UP
microRNA 3656	2.044965985	2.65 × 10^−4^	UP
microRNA 148a	2.072112903	4.99 × 10^−11^	UP
microRNA 4657	2.179581553	2.76 × 10^−2^	UP
microRNA 181b-1	2.338861601	1.59 × 10^−3^	UP
microRNA 181a-1	2.361601036	1.71 × 10^−3^	UP
microRNA 204	3.622749398	2.41 × 10^−30^	UP
microRNA 508	4.093425037	7.10 × 10^−9^	UP
microRNA 155	4.335109793	1.58 × 10^−52^	UP
microRNA 146a	4.399501991	4.11 × 10^−76^	UP
microRNA 509-3	4.724501129	2.50 × 10^−23^	UP
microRNA 509-2	4.959344912	7.21 × 10^−24^	UP
microRNA 509-1	4.959393713	6.68 × 10^−24^	UP

**Table 2:** Demographic and clinical characteristics of melanoma patients.

Case	Sex	Age (Years)	Stage ^a^	BRAF STATUS	Previous Therapy	Targeted Therapy ^c^	BR ^d^	TTF ^e^ (Months)
1	M	46	III–IV	MUT	None	ENCO+BIN	PD	16.90
2	M	42	IV	MUT	None	VEMU	PD	20.83
3	F	50	IV	MUT	None	DAB	PD	9.60
4	M	59	IV	MUT	None	VEMU	PD	18.47
5	M	61	III/IV	MUT	Dacarbazine	VEMU	PD	18.47
6	M	48	NA	MUT	Radiotherapy	VEMU	PR	73.77
7	F	56	IV	MUT	None	VEMU	PD	7.20
8	F	50	III/IV	MUT	None	DAB	PD	15.17
9	M	48	NA	MUT	Radiotherapy	VEMU	PR	73.77
10	M	77	III–IV	MUT	None	DAB + TRAM	PR	43.53
11	F	86	IV	MUT	None	DAB + TRAM	SD	37.23
12	F	70	IV	MUT	None	VEMU + COBI	PR	20.33
13	F	67	III/IV	MUT	None	VEMU + COBI	CR	25.17
14	F	45	NA	MUT	None	DAB + TRAM	PR	21.93
15	M	65	IV	MUT	None	DAB + TRAM	PR	13.43
16	M	47	III/IV	MUT	None	VEMU + COBI	PR	11.53

^a^ Stage at first serum collection (i.e., T0); ^c^ ENCO, encorafenib. BIN, Binimetinib. DAB, dabrafenib. TRAM, trametinib. VEMU, vemurafenib, COBI, cobimetinib; ^d^ Best response at last tumor assessment according to RECIST 1.1 criteria: PR, partial response; SD, stable disease; PD, progressive disease, CR, complete response; ^e^ TTF, time to treatment failure.

**Table 3:** Gene Set Enrichment Analysis (GSEA) querying Hallmark database of DE target genes in melanoma patients expressing high miRNA-181a and -181b versus low.

GENE SET	pval	padj	ES	NES
E2F_TARGETS	0.025	0.067476383	−0.645120028	−2.362223392
MYC_TARGETS_V1	0.025641026	0.067476383	−0.578931346	−2.098724203
MYC_TARGETS_V2	0.005263158	0.029239766	−0.653683002	−2.054477496
G2M_CHECKPOINT	0.025641026	0.067476383	−0.53100629	−1.925856679
UNFOLDED_PROTEIN_RESPONSE	0.010204082	0.051020408	−0.543515092	−1.895494756
MTORC1_SIGNALING	0.025	0.067476383	−0.493595313	−1.804927713
DNA_REPAIR	0.015151515	0.067476383	−0.472340422	−1.677943319
GLYCOLYSIS	0.023255814	0.067476383	−0.443156625	−1.576560747
OXIDATIVE_PHOSPHORYLATION	0.023255814	0.067476383	−0.436113647	−1.557784166
HEDGEHOG_SIGNALING	0.03930131	0.098253275	−0.545687766	−1.479472244
APOPTOSIS	0.050437567	0.153125145	0.480331268	1.267604291
ROS_PATHWAY	0.084367246	0.162244703	0.559128155	1.311901032
P53_PATHWAY	0.003115265	0.019778481	0.529555328	1.416399563
IL2_STAT5_SIGNALING	0.002089864	0.018705574	0.548857874	1.463005267
IL6_JAK_STAT3_SIGNALING	0.001157407	0.014467593	0.649692129	1.607476866
IFNγ_RESPONSE	0.001042753	0.014467593	0.628035534	1.675781046
IFNα_RESPONSE	0.002244669	0.018705574	0.627922857	1.596308497

**Table 4:** Correlation of miR181a and b expression and target genes in melanoma patients.

GENE NAME	cor	pvals
ANKRD13C	−0.574	0.032
ARID2	−0.588	0.027
ARL5A	−0.637	0.014
ATG7	−0.538	0.047
ATL3	−0.535	0.049
CCNK	−0.715	0.004
LRRC32	−0.571	0.033
NAA50	−0.546	0.044
PAWR	−0.586	0.028
PDCD6IP	−0.538	0.047
SLC16A7	−0.565	0.035
SS18L1	−0.575	0.032
TFAM	−0.535	0.049
TMEM134	−0.715	0.004
TMF1	−0.556	0.039
UBP1	−0.696	0.006
ZFP36L1	−0.581	0.029
ZNF791	−0.551	0.041

## Data Availability

The data have been deposited in NCBIs Gene Expression Omnibus (GEO): Superseries GSE165338: RNAseq and Small-RNAseq dataset to comprehensively study the miRNA expression profiling of drug-resistant melanoma patients and cell lines. GSE165334: Transcriptome profile of melanoma cell lines A375 and A375-BIR (BIR-BRAF INHIBITOR RESISTANT) during dabrafenib treatment. GSE165335: SmallRNAome profile of melanoma cell lines A375 and A375-BIR (BIR-BRAF INHIBITOR RESISTANT) during dabrafenib treatment. GSE165337: Transcriptomic profile of melanoma clinical samples treated with BRAF and MEK- inhibitors.

## Supplementary Materials

The following are available online at https://www.mdpi.com/1422-0067/22/4/1801/s1, Figure S1: miR-181a/b Replacement Affects the Growth of Melanoma Cells Sensitive and Resistant Cells to Dabrafenib, Figure S2: miR-181a/b Expression Analysis in Melanoma Patients, Figure S3: GO term enrichment analysis of DE miRNA-181a and -181b-predicted target genes between A375 and A375-BIR, Table S1: Differentially expressed miRNA181a/b-target genes between A375 and A375-BIR cell lines, Table S2: Functional annotation of target genes negatively regulated by miRNA 181a/b in melanoma.

## References

[1] Fischer G.M., Gopal Y.N.V., McQuade J.L., Peng W., DeBerardinis R.J., Davies M.A. (2017). Metabolic strategies of melanoma cells: Mechanisms, interactions with the tumor microenvironment, and therapeutic implications. Pigment. Cell Melanoma Res..

[2] Rajkumar S., Watson I.R. (2016). Molecular characterisation of cutaneous melanoma: Creating a framework for targeted and immune therapies. Br. J. Cancer.

[3] Paluncic J., Kovacevic Z., Jansson P.J., Kalinowski D., Merlot A.M., Huang M.L.-H., Lok H.C., Sahni S., Lane D.J., Richardson D.R. (2016). Roads to melanoma: Key pathways and emerging players in melanoma progression and oncogenic signaling. Biochim. et Biophys. Acta BBA Bioenerg..

[4] Luke J.J., Flaherty K.T., Ribas A., Long G.V. (2017). Targeted agents and immunotherapies: Optimizing outcomes in melanoma. Nat. Rev. Clin. Oncol..

[5] Sullivan R.J., Flaherty K.T. (2013). Resistance to BRAF-targeted therapy in melanoma. Eur. J. Cancer.

[6] Kozar I., Margue C., Rothengatter S., Haan C., Kreis S. (2019). Many ways to resistance: How melanoma cells evade targeted therapies. Biochim. et Biophys. Acta (BBA) Bioenerg..

[7] Gide T.N., Wilmott J.S., Scolyer R.A., Long G.V. (2018). Primary and Acquired Resistance to Immune Checkpoint Inhibitors in Metastatic Melanoma. Clin. Cancer Res..

[8] Fattore L., Costantini S., Malpicci D., Ruggiero C.F., Ascierto P.A., Croce C.M., Mancini R., Ciliberto G. (2017). MicroRNAs in melanoma development and resistance to target therapy. Oncotarget.

[9] Stark M.S., Tyagi S., Nancarrow D.J., Boyle G.M., Cook A.L., Whiteman D.C., Parsons P.G., Schmidt C., Sturm R.A., Hayward N.K. (2010). Characterization of the Melanoma miRNAome by Deep Sequencing. PLoS ONE.

[10] Voortman J., Goto A., Mendiboure J., Sohn J.J., Schetter A.J., Saito M., Dunant A., Pham T.C., Petrini I., Lee A. (2010). MicroRNA Expression and Clinical Outcomes in Patients Treated with Adjuvant Chemotherapy after Complete Resection of Non–Small Cell Lung Carcinoma. Cancer Res..

[11] Previdi M.C., Carotenuto P., Zito D., Pandolfo R., Braconi C. (2017). Noncoding RNAs as novel biomarkers in pancreatic cancer: What do we know?. Futur. Oncol..

[12] Carotenuto P., Hedayat S., Fassan M., Cardinale V., Lampis A., Guzzardo V., Vicentini C., Scarpa A., Cascione L., Costantini D. (2020). Modulation of Biliary Cancer Chemo-Resistance through MicroRNA-Mediated Rewiring of the Expansion of CD133+ Cells. Hepatology.

[13] Indrieri A., Carrella S., Carotenuto P., Banfi S., Franco B. (2020). The Pervasive Role of the miR-181 Family in Development, Neurodegeneration, and Cancer. Int. J. Mol. Sci..

[14] Miller T.E., Ghoshal K., Ramaswamy B., Roy S., Datta J., Shapiro C.L., Jacob S., Majumder S. (2008). MicroRNA-221/222 confers tamoxifen resistance in breast cancer by targeting p27Kip1. J. Biol. Chem..

[15] Liu J., Xing Y., Rong L. (2018). miR-181 regulates cisplatin-resistant non-small cell lung cancer via downregulation of autophagy through the PTEN/PI3K/AKT pathway. Oncol. Rep..

[16] Nakajima G., Hayashi K., Xi Y., Kudo K., Uchida K., Takasaki K., Yamamoto M., Ju J. (2006). Non-coding microRNAs hsa-let-7g and hsa-miR-181b are associated with chemoresponse to S-1 in colon cancer. Cancer Genom. Proteom..

[17] Hasegawa S., Eguchi H., Nagano H., Konno M., Tomimaru Y., Wada H., Hama N., Kawamoto K., Kobayashi S., Nishida N. (2014). MicroRNA-1246 expression associated with CCNG2-mediated chemoresistance and stemness in pancreatic cancer. Br. J. Cancer.

[18] Caporali S., Amaro A., Levati L., Alvino E., Lacal P.M., Mastroeni S., Ruffini F., Bonmassar L., Cappellini G.C.A., Felli N. (2019). miR-126-3p down-regulation contributes to dabrafenib acquired resistance in melanoma by up-regulating ADAM9 and VEGF-A. J. Exp. Clin. Cancer Res..

[19] Fattore L., Mancini R., Acunzo M., Romano G., Laganà A., Pisanu M.E., Malpicci D., Madonna G., Mallardo D., Caponea M. (2016). miR-579-3p controls melanoma progression and resistance to target therapy. Proc. Natl. Acad. Sci. USA.

[20] Lunavat T.R., Cheng L., Einarsdottir B.O., Bagge R.O., Muralidharan S.V., Sharples R.A., Lässer C., Gho Y.S., Hill A.F., Nilsson J.A. (2017). BRAFV600 inhibition alters the microRNA cargo in the vesicular secretome of malignant melanoma cells. Proc. Natl. Acad. Sci. USA.

[21] Patil S.L., Palat A., Pan Y., Rajapakshe K., Mirchandani R., Bondesson M., Yustein J.T., Coarfa C., Gunaratne P.H. (2019). MicroRNA-509-3p inhibits cellular migration, invasion, and proliferation, and sensitizes osteosarcoma to cisplatin. Sci. Rep..

[22] Zhou B., Li C., Jing Q., Liu M.F., Zhai Q., Qi W., Zhang Y., Zhang F., Wu J.X., Hu Y.N. (2012). Downregulation of miR-181a upregulates sirtuin-1 (SIRT1) and improves hepatic insulin sensitivity. Diabetologia.

[23] Xiang Z., Dong X., Sun Q., Li X., Yan B. (2014). Clinical significance of up-regulated miR-181a in prognosis and progression of esophageal cancer. Acta Biochim. et Biophys. Sin..

[24] He S., Zeng S., Zhou Z.W., He Z.X., Zhou S.F. (2015). Hsa-microRNA-181a is a regulator of a number of cancer genes and a biomarker for endometrial carcinoma in patients: A bioinformatic and clinical study and the therapeutic implication. Drug Des. Devel. Ther..

[25] Ebert M.S., Sharp P.A. (2010). MicroRNA sponges: Progress and possibilities. RNA.

[26] Tay F.C., Lim J.K., Zhu H., Hin L.C., Wang S. (2015). Using artificial microRNA sponges to achieve microRNA loss-of-function in cancer cells. Adv. Drug Deliv. Rev..

[27] Indrieri A., Grimaldi C., Zucchelli S., Tammaro R., Gustincich S., Franco B. (2016). Synthetic long non-coding RNAs [SINEUPs] rescue defective gene expression in vivo. Sci. Rep..

[28] Tan J.Y.L., Habib N.A., Chuah Y.W., Yau Y.H., Geifman-Shochat S., Chen W.N. (2015). Identification of Cellular Targets of MicroRNA-181a in HepG2 Cells: A New Approach for Functional Analysis of MicroRNAs. PLoS ONE.

[29] Song J., Yang S., Yin R., Xiao Q., Ma A., Pan X. (2019). MicroRNA-181a regulates the activation of the NLRP3 inflammatory pathway by targeting MEK1 in THP-1 macrophages stimulated by ox-LDL. J. Cell. Biochem..

[30] Li S., Yang J., Xia Y., Fan Q., Yang K.-P. (2018). Long Noncoding RNA NEAT1 Promotes Proliferation and Invasion via Targeting miR-181a-5p in Non-Small Cell Lung Cancer. Oncol. Res. Featur. Preclin. Clin. Cancer Ther..

[31] Araujo L.F., Siena A.D.D., Plaça J.R., Brotto D.B., Barros I.I., Muys B.R., Biagi C.A.O., Peronni K.C., Sousa J.F., Molfetta G.A. (2018). Mitochondrial transcription factor A (TFAM) shapes metabolic and invasion gene signatures in melanoma. Sci. Rep..

[32] Moriceau G., Hugo W., Hong A., Shi H., Kong X., Yu C.C., Koya R.C., Samatar A.A., Khanlou N., Braun J. (2015). Tunable-Combinatorial Mechanisms of Acquired Resistance Limit the Efficacy of BRAF/MEK Cotargeting but Result in Melanoma Drug Addiction. Cancer Cell.

[33] Roesch A., Paschen A., Landsberg J., Helfrich I., Becker J.C., Schadendorf D. (2016). Phenotypic tumour cell plasticity as a resistance mechanism and therapeutic target in melanoma. Eur. J. Cancer.

[34] Mueller D.W., Rehli M., Bosserhoff A.K. (2009). miRNA Expression Profiling in Melanocytes and Melanoma Cell Lines Reveals miRNAs Associated with Formation and Progression of Malignant Melanoma. J. Investig. Dermatol..

[35] Chakraborty C., Sharma A.R., Sharma G., Doss C.G.P., Lee S.-S. (2017). Therapeutic miRNA and siRNA: Moving from Bench to Clinic as Next Generation Medicine. Mol. Ther. Nucleic Acids.

[36] Rupaimoole R., Slack F.J. (2017). MicroRNA therapeutics: Towards a new era for the management of cancer and other diseases. Nat. Rev. Drug Discov..

[37] Li X.J., Ren Z.J., Tang J.H. (2014). MicroRNA-34a: A potential therapeutic target in human cancer. Cell Death Dis..

[38] Ouyang M., Li Y., Ye S., Ma J., Lu L., Lv W., Chang G., Li X., Li Q., Wang S. (2014). MicroRNA Profiling Implies New Markers of Chemoresistance of Triple-Negative Breast Cancer. PLoS ONE.

[39] Chen Y., Ke G., Han D., Liang S., Yang G., Wu X. (2014). MicroRNA-181a enhances the chemoresistance of human cervical squamous cell carcinoma to cisplatin by targeting PRKCD. Exp. Cell Res..

[40] Jornayvaz F.R., Shulman G.I. (2010). Regulation of mitochondrial biogenesis. Essays Biochem..

[41] Zhang G., Frederick D.T., Wu L., Wei Z., Krepler C., Srinivasan S., Chae Y.C., Xu X., Choi H., Dimwamwa E. (2016). Targeting mitochondrial biogenesis to overcome drug resistance to MAPK inhibitors. J. Clin. Investig..

[42] Indrieri A., Carrella S., Romano A., Spaziano A., Marrocco E., Fernandez-Vizarra E., Barbato S., Pizzo M., Ezhova Y., Golia F.M. (2019). miR-181a/b downregulation exerts a protective action on mitochondrial disease models. EMBO Mol. Med..

[43] Sun X., Li J., Sun Y., Zhang Y., Dong L., Shen C., Yang L., Yang M., Li Y., Shen G. (2016). miR-7 reverses the resistance to BRAFi in melanoma by targeting EGFR/IGF-1R/CRAF and inhibiting the MAPK and PI3K/AKT signaling pathways. Oncotarget.

[44] Koetz-Ploch L., Hanniford U., Dolgalev I., Sokolova E., Zhong J., Díaz-Martínez M., Bernstein E., Darvishian F., Flaherty K.T., Chapman P.B. (2017). MicroRNA-125a promotes resistance to BRAF inhibitors through suppression of the intrinsic apoptotic pathway. Pigment. Cell Melanoma Res..

[45] de Filippis D., Russo A., de Stefano D., Cipriano M., Esposito D., Grassia G., Carnuccio R., Russo G., Iuvone T. (2014). Palmitoylethanolamide inhibits rMCP-5 expression by regulating MITF activation in rat chronic granulomatous inflammation. Eur. J. Pharmacol..

[46] Lampis A., Carotenuto P., Vlachogiannis G., Cascione L., Hedayat S., Burke R., Clarke P.A., Bosma E., Simbolo M., Scarpa A. (2018). MIR21 Drives Resistance to Heat Shock Protein 90 Inhibition in Cholangiocarcinoma. Gastroenterology.

[47] Pecoraro A., Carotenuto P., Franco B., De Cegli R., Russo G., Russo A. (2020). Role of uL3 in the Crosstalk between Nucleolar Stress and Autophagy in Colon Cancer Cells. Int. J. Mol. Sci..

[48] Robinson M.D., Oshlack A. (2010). A scaling normalization method for differential expression analysis of RNA-seq data. Genome Biol..

