# Supplementary material for: Integrated Genomics Identifies miR-181/TFAM Pathway as a Critical Driver of Drug Resistance in Melanoma
Source: Int J Mol Sci. 2021 Feb 11;22(4):1801. doi: 10.3390/ijms22041801 (PMC7918089; doi:10.3390/ijms22041801)
Supplement: Supplementary file 1 [file ijms-22-01801-s001.pdf]

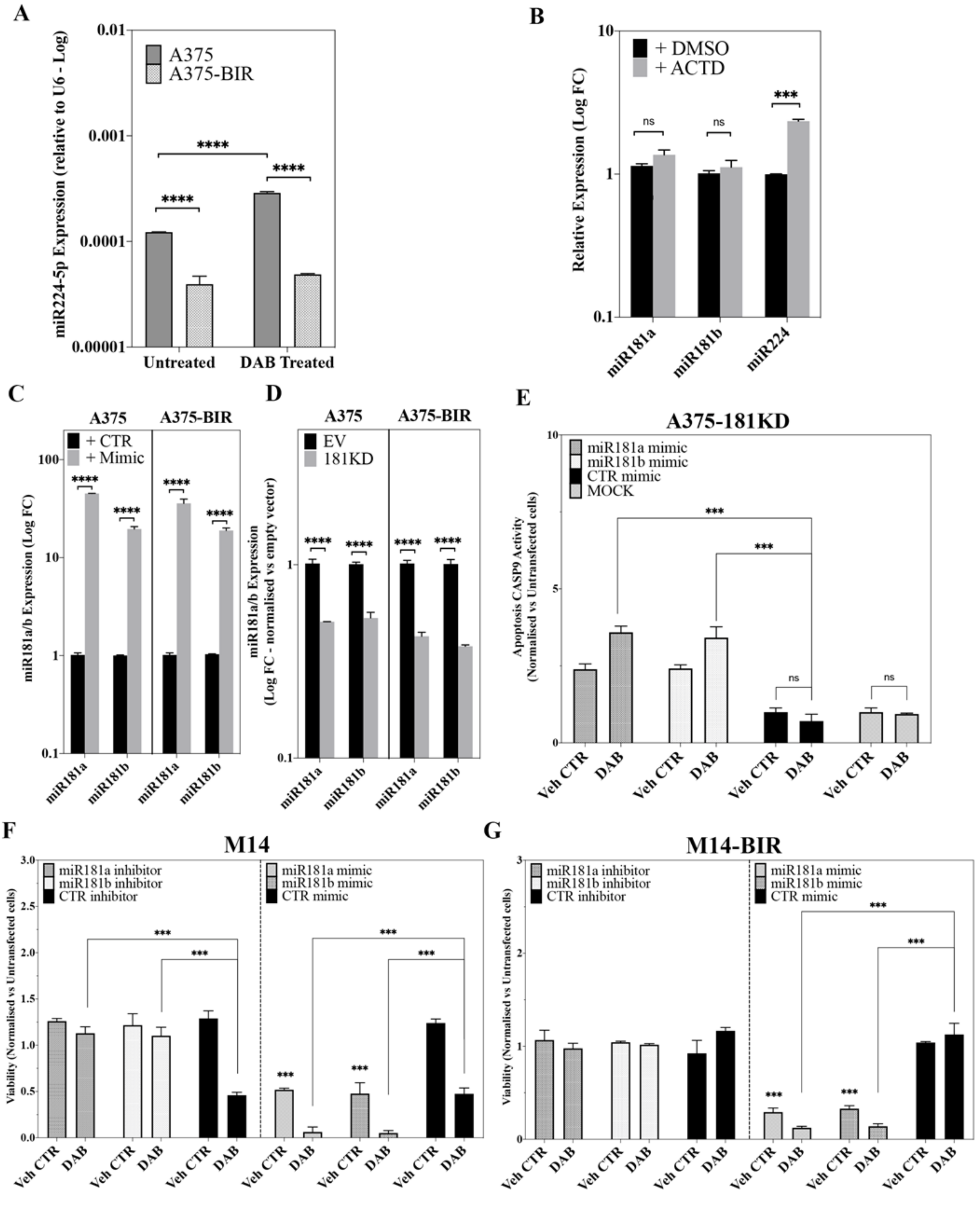

Supplementary Figure S1. A) The expression levels of miR-224 in A375, A375-BIR melanoma cells, measured by qRT-PCR. **B)** The expression levels of miR-181a, -181b and miR-22a in A375, melanoma cells, treated with 5nM Actinomycin D (ACTD) and vehicle control (DMSO), measured by qRT-PCR. The miRNA expression levels were normalized to the internal control U6. ***p < 0.001; ns, not significatively. **C)** The expression levels of miR-181a, -181b in A375 and A375-BIR melanoma cells transfected with mimic and CTR; **D)** The expression levels of miR-181a, -181b in A375 and A375-BIR

melanoma cells transfected with miR181a/b sponge (181KD) and pcDNA3.1-GFP used as empty vector CTR; **E)** Effect of miRNA-181a and b forced expression or inhibition on CASP-9 activation in A375-181KD cells. The determination of CASP-9 activity was carried out by using Caspase-Glo® 9 assay. Data are expressed in fold change relative to vehicle control +/-SD of three independent assays with 3 replicates each one. ***P<0.001, and *P<0.01 *versus* vehicle control; ns, not significative; **F-G)** M14 **(F)** and M14-BIR **(G)** cells transiently transfected with 40 nM of the miR-181a and -181b mimic or inhibitor or the appropriate controls were treated with 200nM dabrafenib and assayed for proliferation by the MTT assay. Data are expressed in terms of percentage of cell viability as compared to untransfected cells. Each value represents the arithmetic mean of three independent experiments performed with triplicate samples. ***P<0.001; *P<0.01.

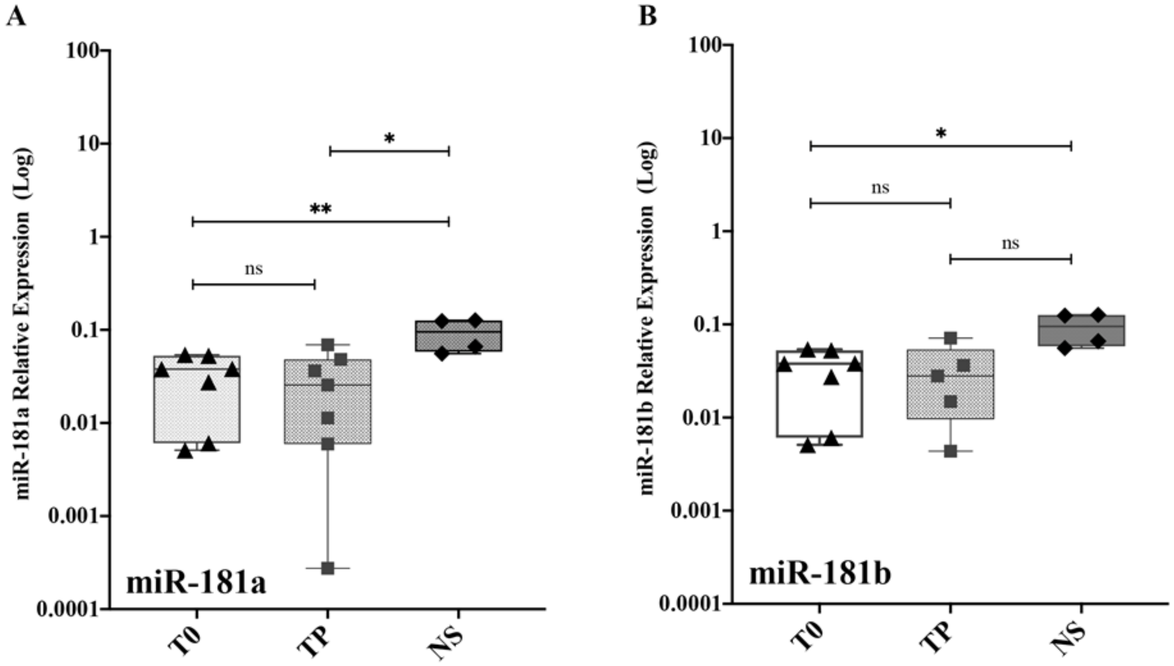

Supplementary Figure S2. A-B) Box-and-whisker diagrams of miRNA-181a (A) and -181b (B) levels in melanoma patients before the start of therapy (pre-treatment), after treatment (post-treatment) and in normal skin sample. The horizontal bar within each box indicates the median. Data were analysed by non-parametric Wilcoxon matched-pairs signed-rank test. **P<0.001; *P<0.01; ns = not significative.

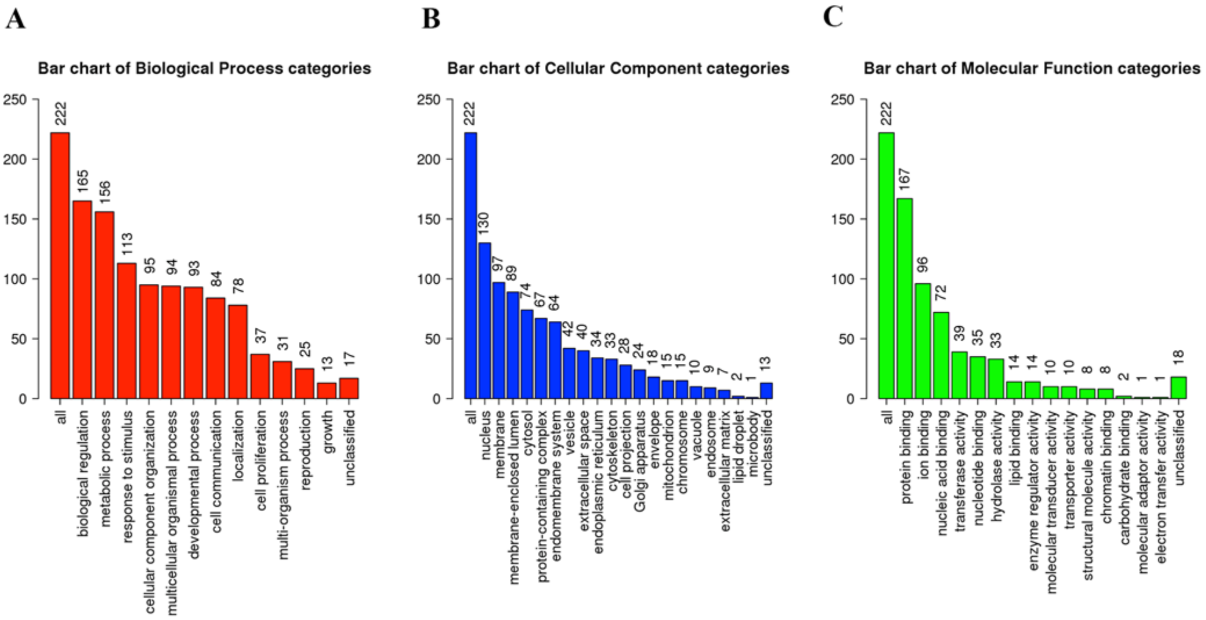

Supplementary Figure S3. A-B-C) GO term enrichment analysis of DE miRNA-181a and -181b-predicted target genes between A375 and A375-BIR. Bar plots shows the distribution of GO Biological Process Gene set (A), GO Cellular Component (B), Molecular Function (C) ranked by gene number.

Supplemental Table S1. Differentially expressed miRNA181a/b-target genes between A375 and A375-BIR cell lines.

Gene Name	EnsemblGeneID	logFC	Pvalue	FDR	regulation	Representative miRNA
LIMCH1	ENSG00000064042	-4.892335943	1.75631E-68	3.45889E-66	DOWN	hsa-miR-181a-5p
HS3ST3A1	ENSG00000153976	-6.778414602	2.14351E-66	3.89672E-64	DOWN	hsa-miR-181a-5p
PAWR	ENSG00000177425	-2.208172689	7.96001E-50	8.00406E-48	DOWN	hsa-miR-181a-5p
COL5A1	ENSG00000130635	-8.506989947	4.05663E-40	2.8557E-38	DOWN	hsa-miR-181a-5p
BMPR2	ENSG00000204217	-2.765427956	6.76237E-33	3.37975E-31	DOWN	hsa-miR-181a-5p
LBR	ENSG00000143815	-2.041225474	2.04311E-28	7.56134E-27	DOWN	hsa-miR-181b-5p
CCNG1	ENSG00000113328	-2.336311377	7.0267E-26	2.25714E-24	DOWN	hsa-miR-181b-5p
MPP5	ENSG00000072415	-1.645318721	9.4931E-25	2.80436E-23	DOWN	hsa-miR-181a-5p
CNKSR2	ENSG00000149970	-4.302359101	1.9331E-23	5.25975E-22	DOWN	hsa-miR-181b-5p
SLC16A7	ENSG00000118596	-1.630638997	6.97174E-23	1.79089E-21	DOWN	hsa-miR-181a-5p
TM9SF3	ENSG00000077147	-1.271388243	2.14836E-22	5.34442E-21	DOWN	hsa-miR-181a-5p
ONECUT2	ENSG00000119547	-4.59577696	1.56087E-21	3.67304E-20	DOWN	hsa-miR-181a-5p
HMGB2	ENSG00000164104	-1.505499462	1.00485E-19	2.07531E-18	DOWN	hsa-miR-181a-5p
SCD	ENSG00000099194	-1.793560571	1.3231E-17	2.30401E-16	DOWN	hsa-miR-181a-5p
FIGN	ENSG00000182263	-2.376542174	1.22154E-16	1.98702E-15	DOWN	hsa-miR-181a-5p
TSC22D2	ENSG00000196428	-1.827370225	1.94851E-16	3.11442E-15	DOWN	hsa-miR-181a-5p
TFAM	ENSG00000108064	-1.229275427	9.74288E-16	1.4814E-14	DOWN	hsa-miR-181b-5p
LIN28B	ENSG00000187772	-5.442900493	6.6211E-15	9.1736E-14	DOWN	hsa-miR-181a-5p
EXT1	ENSG00000182197	-1.602788385	1.26763E-14	1.71326E-13	DOWN	hsa-miR-181a-5p
ZNF33B	ENSG00000196693	-2.046951807	3.55692E-13	4.27321E-12	DOWN	hsa-miR-181a-5p
GHITM	ENSG00000165678	-1.199822632	4.80712E-13	5.69658E-12	DOWN	hsa-miR-181a-5p
SRSF7	ENSG00000115875	-1.190101567	6.26E-13	7.3135E-12	DOWN	hsa-miR-181a-5p
VCAN	ENSG00000038427	-1.305481435	6.3963E-13	7.46023E-12	DOWN	hsa-miR-181a-5p
PCNP	ENSG00000081154	-1.124780814	1.16704E-12	1.32598E-11	DOWN	hsa-miR-181a-5p
OGT	ENSG00000147162	-1.818760551	2.69423E-12	2.92843E-11	DOWN	hsa-miR-181a-5p
GNAQ	ENSG00000156052	-1.048377924	5.39888E-12	5.71059E-11	DOWN	hsa-miR-181a-5p
NEK7	ENSG00000151414	-1.645883549	1.40919E-11	1.41888E-10	DOWN	hsa-miR-181a-5p
ARL5A	ENSG00000162980	-1.165455656	2.63661E-10	2.3005E-09	DOWN	hsa-miR-181a-5p
CREBZF	ENSG00000137504	-1.068320616	3.29854E-10	2.84355E-09	DOWN	hsa-miR-181a-5p
PHF3	ENSG00000118482	-0.914596443	1.29688E-09	1.044E-08	DOWN	hsa-miR-181a-5p
SLITRK1	ENSG00000178235	-6.336558584	2.22561E-09	1.73835E-08	DOWN	hsa-miR-181b-5p

AHNAK	ENSG00000124942	-0.925752413	2.71715E-09	2.09459E-08	DOWN	hsa-miR-181a-5p
PDCD6IP	ENSG00000170248	-0.988862982	3.07228E-09	2.34756E-08	DOWN	hsa-miR-181a-5p
MAP2K1	ENSG00000169032	-2.178695479	3.12974E-09	2.38706E-08	DOWN	hsa-miR-181a-5p
PBX1	ENSG00000185630	-6.617336657	5.70747E-09	4.20386E-08	DOWN	hsa-miR-181a-5p
PARM1	ENSG00000169116	-3.878453652	6.56394E-09	4.8005E-08	DOWN	hsa-miR-181a-5p
ARL3	ENSG00000138175	-1.130402403	8.39941E-09	6.08902E-08	DOWN	hsa-miR-181a-5p
MBOAT2	ENSG00000143797	-0.908716715	9.58399E-09	6.90841E-08	DOWN	hsa-miR-181a-5p
NIPBL	ENSG00000164190	-1.053505701	1.47346E-08	1.03947E-07	DOWN	hsa-miR-181a-5p
ZNF283	ENSG00000167637	-1.620418794	2.41763E-08	1.66299E-07	DOWN	hsa-miR-181a-5p
ELAVL2	ENSG00000107105	-5.832120127	2.5428E-08	1.74257E-07	DOWN	hsa-miR-181a-5p
RIMKLB	ENSG00000166532	-1.452518261	6.03138E-08	3.92052E-07	DOWN	hsa-miR-181a-5p
UBE2W	ENSG00000104343	-1.283063457	6.31976E-08	4.09349E-07	DOWN	hsa-miR-181a-5p
DUSP5	ENSG00000138166	-1.245675844	6.32701E-08	4.09659E-07	DOWN	hsa-miR-181a-5p
MYBL1	ENSG00000185697	-0.841747723	7.08628E-08	4.54902E-07	DOWN	hsa-miR-181a-5p
LYRM1	ENSG00000102897	-1.890940993	7.56963E-08	4.84453E-07	DOWN	hsa-miR-181a-5p
HNRNPR	ENSG00000125944	-0.788562279	9.4723E-08	5.98093E-07	DOWN	hsa-miR-181b-5p
THRB	ENSG00000151090	-1.979534898	9.98892E-08	6.29747E-07	DOWN	hsa-miR-181a-5p
TRNP1	ENSG00000253368	-1.086599175	1.4659E-07	9.01839E-07	DOWN	hsa-miR-181a-5p
SAMHD1	ENSG00000101347	-1.429199756	3.25149E-07	1.89399E-06	DOWN	hsa-miR-181a-5p
YOD1	ENSG00000180667	-1.890981939	3.84324E-07	2.21915E-06	DOWN	hsa-miR-181a-5p
ADAMTS5	ENSG00000154736	-3.27033551	4.21426E-07	2.41067E-06	DOWN	hsa-miR-181a-5p
ZNF397	ENSG00000186812	-1.18742709	5.63732E-07	3.15488E-06	DOWN	hsa-miR-181a-5p
FAM19A2	ENSG00000198673	-1.995559751	7.79024E-07	4.2617E-06	DOWN	hsa-miR-181a-5p
TGFBR1	ENSG00000106799	-1.6874707	8.22208E-07	4.48607E-06	DOWN	hsa-miR-181b-5p
PRPF40A	ENSG00000196504	-0.577612056	8.38038E-07	4.56492E-06	DOWN	hsa-miR-181a-5p
RORB	ENSG00000198963	-3.319796576	9.19695E-07	4.98346E-06	DOWN	hsa-miR-181a-5p
PDCD4	ENSG00000150593	-1.125899036	1.18805E-06	6.32566E-06	DOWN	hsa-miR-181a-5p
ETS1	ENSG00000134954	-0.95375958	1.24007E-06	6.57306E-06	DOWN	hsa-miR-181a-5p
RORA	ENSG00000069667	-1.429349405	1.56704E-06	8.18291E-06	DOWN	hsa-miR-181a-5p
AFF1	ENSG00000172493	-2.805671516	2.01167E-06	1.03159E-05	DOWN	hsa-miR-181a-5p
CAND1	ENSG00000111530	-0.694653319	3.62783E-06	1.7867E-05	DOWN	hsa-miR-181a-5p
ANKRD13C	ENSG00000118454	-1.538794104	4.27345E-06	2.07671E-05	DOWN	hsa-miR-181a-5p
PNISR	ENSG00000132424	-1.003934423	5.17441E-06	2.49055E-05	DOWN	hsa-miR-181a-5p
DDX52	ENSG00000278053	-0.736751213	5.90061E-06	2.80984E-05	DOWN	hsa-miR-181a-5p
SMAD2	ENSG00000175387	-0.747935495	7.90833E-06	3.68944E-05	DOWN	hsa-miR-181a-5p
KPNA4	ENSG00000186432	-0.593409129	1.24126E-05	5.61042E-05	DOWN	hsa-miR-181a-5p
MAPK8	ENSG00000107643	-1.298354591	1.57507E-05	7.00442E-05	DOWN	hsa-miR-181a-5p
FBXL3	ENSG00000005812	-1.906359667	2.03389E-05	8.86136E-05	DOWN	hsa-miR-181a-5p
SIAE	ENSG00000110013	-1.290672909	3.14379E-05	0.000132134	DOWN	hsa-miR-181a-5p
OSBPL8	ENSG00000091039	-0.617480744	3.24496E-05	0.000136178	DOWN	hsa-miR-181a-5p
ZNF268	ENSG00000090612	-1.245041076	3.34151E-05	0.000139786	DOWN	hsa-miR-181a-5p
SLC35E1	ENSG00000127526	-0.632351514	3.79774E-05	0.000157065	DOWN	hsa-miR-181a-5p
AK3	ENSG00000147853	-0.637241611	4.12564E-05	0.000169398	DOWN	hsa-miR-181a-5p
UBP1	ENSG00000153560	-0.748481233	4.24062E-05	0.000173645	DOWN	hsa-miR-181a-5p
EXOSC3	ENSG00000107371	-1.371786472	6.03417E-05	0.000240017	DOWN	hsa-miR-181a-5p
QKI	ENSG00000112531	-0.689135341	6.08129E-05	0.000241485	DOWN	hsa-miR-181a-5p
E2F7	ENSG00000165891	-0.850226841	9.60042E-05	0.000367129	DOWN	hsa-miR-181a-5p
ARL1	ENSG00000120805	-0.581958384	0.000134036	0.000498507	DOWN	hsa-miR-181a-5p
GREB1L	ENSG00000141449	-1.298909662	0.000137707	0.000510667	DOWN	hsa-miR-181a-5p
MELK	ENSG00000165304	-1.322975538	0.000142146	0.000526066	DOWN	hsa-miR-181a-5p
C14orf28	ENSG00000179476	-4.736785219	0.000205424	0.000737803	DOWN	hsa-miR-181a-5p
BIRC6	ENSG00000115760	-0.609950485	0.000235572	0.000835204	DOWN	hsa-miR-181a-5p
ZFP36L2	ENSG00000152518	-0.803477461	0.00023576	0.000835692	DOWN	hsa-miR-181a-5p
ADAT2	ENSG00000189007	-0.933322433	0.000280038	0.000977355	DOWN	hsa-miR-181a-5p
NUDT21	ENSG00000167005	-0.64820926	0.00028694	0.000999968	DOWN	hsa-miR-181b-5p
SLC35G1	ENSG00000176273	-1.249834096	0.000369898	0.001264826	DOWN	hsa-miR-181a-5p
BTG2	ENSG00000159388	-1.542476348	0.000411555	0.001392303	DOWN	hsa-miR-181a-5p
PAX9	ENSG00000198807	-2.236854897	0.000477596	0.001591314	DOWN	hsa-miR-181a-5p
CYLD	ENSG00000083799	-0.811983572	0.000508894	0.001683936	DOWN	hsa-miR-181a-5p
DNAJB14	ENSG00000164031	-0.620000128	0.000536383	0.001763741	DOWN	hsa-miR-181a-5p
IARS2	ENSG00000067704	-0.729843102	0.000556254	0.001822561	DOWN	hsa-miR-181a-5p
RASSF1	ENSG00000068028	-1.162325239	0.000561251	0.001836386	DOWN	hsa-miR-181a-5p
ARSJ	ENSG00000180801	-1.014506292	0.000610609	0.001984148	DOWN	hsa-miR-181a-5p

CCAR1	ENSG00000060339	-0.762830687	0.000637796	0.002065594	DOWN	hsa-miR-181a-5p
DCUN1D1	ENSG00000043093	-0.921907234	0.000645488	0.002088871	DOWN	hsa-miR-181a-5p
MOSPD1	ENSG00000101928	-1.106041237	0.000665119	0.002147779	DOWN	hsa-miR-181a-5p
ANKFY1	ENSG00000185722	-0.773256383	0.00077027	0.002453325	DOWN	hsa-miR-181a-5p
IPPK	ENSG00000127080	-1.355114065	0.000781392	0.002485402	DOWN	hsa-miR-181a-5p
WDFY3	ENSG00000163625	-1.007679657	0.000963008	0.002999823	DOWN	hsa-miR-181a-5p
LMBRD2	ENSG00000164187	-0.6589327	0.00108834	0.003352152	DOWN	hsa-miR-181a-5p
USP47	ENSG00000170242	-0.521047543	0.001334158	0.004029099	DOWN	hsa-miR-181a-5p
SCOC	ENSG00000153130	-0.578951579	0.001380351	0.004157892	DOWN	hsa-miR-181a-5p
TTLL7	ENSG00000137941	-0.897900825	0.001383309	0.004165287	DOWN	hsa-miR-181a-5p
CYP26B1	ENSG00000003137	-4.128100732	0.001401248	0.004211635	DOWN	hsa-miR-181a-5p
USP33	ENSG00000077254	-0.441431181	0.00167095	0.004902895	DOWN	hsa-miR-181a-5p
SMAP1	ENSG00000112305	-0.604182863	0.001781628	0.005194479	DOWN	hsa-miR-181a-5p
CDH8	ENSG00000150394	-3.285202672	0.002063629	0.005931992	DOWN	hsa-miR-181a-5p
MIER3	ENSG00000155545	-1.066477131	0.002184561	0.006238425	DOWN	hsa-miR-181a-5p
PNRC1	ENSG00000146278	-0.75097866	0.002273858	0.006462194	DOWN	hsa-miR-181a-5p
ZFP30	ENSG00000120784	-1.39866113	0.002511546	0.007057669	DOWN	hsa-miR-181a-5p
DEK	ENSG00000124795	-0.400608658	0.002535155	0.007117968	DOWN	hsa-miR-181a-5p
KLHL2	ENSG00000109466	-1.202151843	0.002899202	0.008032405	DOWN	hsa-miR-181a-5p
KLHL5	ENSG00000109790	-0.803006154	0.003382893	0.009205987	DOWN	hsa-miR-181a-5p
ATP1B1	ENSG00000143153	-0.525394267	0.003475992	0.009422142	DOWN	hsa-miR-181a-5p
MLF1	ENSG00000178053	-0.760696719	0.003545472	0.009594756	DOWN	hsa-miR-181b-5p
TNFRSF11B	ENSG00000164761	-3.902731475	0.003877789	0.010383662	DOWN	hsa-miR-181a-5p
BCLAF1	ENSG00000029363	-0.489477862	0.004146608	0.011028511	DOWN	hsa-miR-181a-5p
SIRT1	ENSG00000096717	-1.31470565	0.004203111	0.011157264	DOWN	hsa-miR-181a-5p
CNR1	ENSG00000118432	-2.055247329	0.004719313	0.01238249	DOWN	hsa-miR-181a-5p
LCLAT1	ENSG00000172954	-0.678667271	0.006284576	0.015965256	DOWN	hsa-miR-181a-5p
RAB11A	ENSG00000103769	-0.389587144	0.008814578	0.021406277	DOWN	hsa-miR-181a-5p
ZFP1	ENSG00000184517	-1.094080871	0.00972314	0.023342027	DOWN	hsa-miR-181b-5p
NLK	ENSG00000087095	-0.656526756	0.009802884	0.02348575	DOWN	hsa-miR-181a-5p
RABGEF1	ENSG00000154710	-0.689886204	0.011949868	0.027906078	DOWN	hsa-miR-181a-5p
SIM1	ENSG00000112246	-4.077719764	0.013128184	0.030273146	DOWN	hsa-miR-181a-5p
CCNJ	ENSG00000107443	-1.20313765	0.014504244	0.033081995	DOWN	hsa-miR-181a-5p
CHD1	ENSG00000153922	-0.384467031	0.018589315	0.041074266	DOWN	hsa-miR-181a-5p
CNTN4	ENSG00000144619	-3.404133924	0.019297218	0.042434386	DOWN	hsa-miR-181a-5p
ZFP14	ENSG00000142065	-0.87415752	0.022401092	0.048344034	DOWN	hsa-miR-181a-5p
EYA3	ENSG00000158161	-0.45716329	0.023037013	0.049448722	DOWN	hsa-miR-181a-5p
LRRC8D	ENSG00000171492	-0.606533119	0.023271041	0.04982836	DOWN	hsa-miR-181a-5p
ITGB8	ENSG00000105855	4.11679502	1.99738E-58	2.62244E-56	UP	hsa-miR-181a-5p
QDPR	ENSG00000151552	2.188615561	5.73138E-47	5.04331E-45	UP	hsa-miR-181b-5p
CLASP1	ENSG00000074054	2.574128925	1.59279E-37	9.90586E-36	UP	hsa-miR-181a-5p
ENTPD6	ENSG00000197586	2.45500107	6.80412E-37	4.07828E-35	UP	hsa-miR-181a-5p
TRIM2	ENSG00000109654	2.875650233	4.43541E-35	2.44583E-33	UP	hsa-miR-181a-5p
SPRY4	ENSG00000187678	2.291291849	1.6314E-33	8.40753E-32	UP	hsa-miR-181a-5p
CXADR	ENSG00000154639	2.489368656	7.10772E-31	3.15236E-29	UP	hsa-miR-181a-5p
ARNT2	ENSG00000172379	2.565400227	8.59717E-30	3.52911E-28	UP	hsa-miR-181a-5p
ZKSCAN1	ENSG00000106261	1.393700648	1.42744E-25	4.46393E-24	UP	hsa-miR-181a-5p
CLUH	ENSG00000132361	1.821405239	2.33337E-23	6.2461E-22	UP	hsa-miR-181b-5p
MAP3K3	ENSG00000198909	1.386409569	4.7578E-23	1.24538E-21	UP	hsa-miR-181b-5p
PKNOX2	ENSG00000165495	5.65913103	6.29296E-22	1.51756E-20	UP	hsa-miR-181a-5p
COL16A1	ENSG00000084636	3.227286451	8.21713E-22	1.96723E-20	UP	hsa-miR-181a-5p
NREP	ENSG00000134986	2.218810013	3.94242E-20	8.36146E-19	UP	hsa-miR-181b-5p
MLXIP	ENSG00000175727	1.531710805	1.18148E-17	2.06827E-16	UP	hsa-miR-181a-5p
TANC2	ENSG00000170921	1.851445769	4.33625E-17	7.21676E-16	UP	hsa-miR-181a-5p
BAZ2B	ENSG00000123636	1.455969509	3.18542E-16	5.00441E-15	UP	hsa-miR-181a-5p
LPCAT2	ENSG00000087253	2.110073843	2.60273E-15	3.78024E-14	UP	hsa-miR-181a-5p
SOX5	ENSG00000134532	1.664684799	2.74628E-14	3.6143E-13	UP	hsa-miR-181b-5p
FMNL2	ENSG00000157827	1.010479036	6.58256E-14	8.4219E-13	UP	hsa-miR-181a-5p
UNKL	ENSG00000059145	1.678081426	1.79259E-12	1.99159E-11	UP	hsa-miR-181a-5p
TBC1D4	ENSG00000136111	1.246033295	1.79954E-12	1.99797E-11	UP	hsa-miR-181a-5p
ARHGEF3	ENSG00000163947	1.503979877	3.07601E-12	3.33245E-11	UP	hsa-miR-181a-5p
ANKRD44	ENSG00000065413	2.471876054	2.13181E-11	2.12194E-10	UP	hsa-miR-181a-5p
EPN2	ENSG00000072134	1.147838113	4.81431E-11	4.5851E-10	UP	hsa-miR-181a-5p

AAK1	ENSG00000115977	1.035794111	8.40986E-11	7.77665E-10	UP	hsa-miR-181a-5p
HEY2	ENSG00000135547	3.677318371	1.09098E-10	9.97687E-10	UP	hsa-miR-181a-5p
ZNF697	ENSG00000143067	1.309992037	3.25878E-10	2.81074E-09	UP	hsa-miR-181a-5p
ZNF536	ENSG00000198597	3.280196661	6.65352E-10	5.55905E-09	UP	hsa-miR-181a-5p
CALR	ENSG00000179218	0.852859372	9.4939E-10	7.79829E-09	UP	hsa-miR-181a-5p
GNAO1	ENSG00000087258	1.83437639	1.19328E-09	9.63421E-09	UP	hsa-miR-181a-5p
MAP1A	ENSG00000166963	2.176761633	1.62744E-09	1.29499E-08	UP	hsa-miR-181a-5p
DNAJB1	ENSG00000132002	0.992257414	3.62773E-09	2.74034E-08	UP	hsa-miR-181a-5p
KANK1	ENSG00000107104	1.791456675	4.23303E-09	3.17008E-08	UP	hsa-miR-181a-5p
ZNF652	ENSG00000198740	0.971146023	5.8379E-09	4.29419E-08	UP	hsa-miR-181a-5p
BPTF	ENSG00000171634	0.749707939	1.19431E-08	8.52351E-08	UP	hsa-miR-181a-5p
APLP2	ENSG00000084234	0.782878419	3.30364E-08	2.22344E-07	UP	hsa-miR-181a-5p
CPNE2	ENSG00000140848	1.86246615	7.96971E-08	5.07283E-07	UP	hsa-miR-181a-5p
PDAP1	ENSG00000106244	0.820727341	1.00154E-07	6.30701E-07	UP	hsa-miR-181a-5p
SRPK2	ENSG00000135250	0.739285527	2.12914E-07	1.2771E-06	UP	hsa-miR-181a-5p
GDPD1	ENSG00000153982	1.405684301	2.29679E-07	1.37318E-06	UP	hsa-miR-181a-5p
PIK3R3	ENSG00000117461	1.66620256	3.51106E-07	2.03516E-06	UP	hsa-miR-181a-5p
UBE3C	ENSG00000009335	0.816836209	4.91128E-07	2.77579E-06	UP	hsa-miR-181a-5p
IL1RAPL1	ENSG00000169306	0.918618059	6.02705E-07	3.3537E-06	UP	hsa-miR-181b-5p
FAM222B	ENSG00000173065	0.918525672	6.44457E-07	3.56683E-06	UP	hsa-miR-181a-5p
PDXDC1	ENSG00000179889	0.811855949	1.18775E-06	6.32566E-06	UP	hsa-miR-181b-5p
RNF125	ENSG00000101695	1.711735665	1.73337E-06	8.99189E-06	UP	hsa-miR-181b-5p
TRIM13	ENSG00000204977	0.991341381	2.04993E-06	1.04958E-05	UP	hsa-miR-181a-5p
ZSWIM1	ENSG00000168612	1.856446412	2.41576E-06	1.22439E-05	UP	hsa-miR-181a-5p
PDIA6	ENSG00000143870	0.731822262	2.82354E-06	1.41502E-05	UP	hsa-miR-181a-5p
ELMSAN1	ENSG00000156030	1.004051921	1.21882E-05	5.51502E-05	UP	hsa-miR-181a-5p
TUBD1	ENSG00000108423	1.650157299	2.04642E-05	8.91127E-05	UP	hsa-miR-181a-5p
LIF	ENSG00000128342	3.280555709	2.51628E-05	0.000107869	UP	hsa-miR-181b-5p
RNF8	ENSG00000112130	0.880071915	3.23284E-05	0.000135704	UP	hsa-miR-181b-5p
TUBB	ENSG00000196230	0.759580106	3.83285E-05	0.00015823	UP	hsa-miR-181a-5p
SACM1L	ENSG00000211456	0.841042797	4.51536E-05	0.000183939	UP	hsa-miR-181b-5p
RLF	ENSG00000117000	0.685125252	7.63973E-05	0.000297654	UP	hsa-miR-181a-5p
GSE1	ENSG00000131149	0.875186721	0.000109557	0.000413698	UP	hsa-miR-181b-5p
CKAP4	ENSG00000136026	0.425800343	0.000128658	0.000480448	UP	hsa-miR-181a-5p
PGRMC2	ENSG00000164040	0.728308702	0.000283486	0.000988765	UP	hsa-miR-181a-5p
ZFAND6	ENSG00000086666	0.742098602	0.000328557	0.001134721	UP	hsa-miR-181a-5p
CEP350	ENSG00000135837	0.478703072	0.000439451	0.00147791	UP	hsa-miR-181a-5p
TMCC1	ENSG00000172765	0.664020688	0.000441756	0.001484456	UP	hsa-miR-181b-5p
ACVR1C	ENSG00000123612	2.040718863	0.000479588	0.001596984	UP	hsa-miR-181a-5p
PSPC1	ENSG00000121390	0.8472819	0.000598975	0.001949026	UP	hsa-miR-181a-5p
PRDM4	ENSG00000110851	0.684679525	0.000732263	0.002346732	UP	hsa-miR-181a-5p
SERTAD4	ENSG00000082497	0.536522245	0.001054932	0.00325957	UP	hsa-miR-181a-5p
RAB8B	ENSG00000166128	0.817779824	0.001306916	0.003955416	UP	hsa-miR-181a-5p
ZCCHC14	ENSG00000140948	1.147409318	0.001679727	0.004927776	UP	hsa-miR-181a-5p
SLC44A1	ENSG00000070214	0.414116406	0.001889162	0.005480955	UP	hsa-miR-181a-5p
SBNO1	ENSG00000139697	0.487773891	0.001976197	0.00570218	UP	hsa-miR-181a-5p
DNAJC5	ENSG00000101152	0.523885569	0.002128589	0.006094366	UP	hsa-miR-181a-5p
PRKCD	ENSG00000163932	0.686478865	0.002456459	0.006921684	UP	hsa-miR-181a-5p
IGSF11	ENSG00000144847	4.265701215	0.002484424	0.006992145	UP	hsa-miR-181a-5p
MCL1	ENSG00000143384	0.417887978	0.002875235	0.007978031	UP	hsa-miR-181a-5p
MYO1E	ENSG00000157483	0.550687761	0.003282618	0.008965552	UP	hsa-miR-181a-5p
OSBPL3	ENSG00000070882	0.595546174	0.004563892	0.01201854	UP	hsa-miR-181b-5p
SEC24C	ENSG00000176986	0.801493454	0.004915127	0.01284533	UP	hsa-miR-181a-5p
FAM160A2	ENSG00000051009	1.009536397	0.0066642	0.01680065	UP	hsa-miR-181a-5p
KMT2E	ENSG00000005483	0.35116421	0.007095418	0.017779384	UP	hsa-miR-181a-5p
WSB2	ENSG00000176871	0.771362728	0.007173626	0.017921065	UP	hsa-miR-181a-5p
LRRC32	ENSG00000137507	4.196658545	0.008107477	0.019902358	UP	hsa-miR-181a-5p
B3GALT1	ENSG00000172318	2.322533622	0.009626309	0.023136426	UP	hsa-miR-181b-5p
MOB3B	ENSG00000120162	0.871244588	0.011015873	0.026033655	UP	hsa-miR-181a-5p
SEMA4F	ENSG00000135622	1.077324979	0.015853953	0.035731872	UP	hsa-miR-181a-5p
MAPRE2	ENSG00000166974	0.507593429	0.016724638	0.037423136	UP	hsa-miR-181a-5p
ZDHHC7	ENSG00000153786	0.719411413	0.019015102	0.041880819	UP	hsa-miR-181a-5p
RNF34	ENSG00000170633	0.504165776	0.019106356	0.042048789	UP	hsa-miR-181b-5p

STAT3	ENSG00000168610	0.451031614	0.019288654	0.042426832	UP	hsa-miR-181b-5p
TRIP4	ENSG00000103671	0.926371266	0.019312397	0.042450834	UP	hsa-miR-181a-5p

Supplemental Table S2. Functional annotation of target genes negatively regulated by miRNA 181a/b in melanoma.

Functional Category	Enrichment FDR	Genes in list	Genes
Regulation of biosynthetic process	0.017090142	11	CCNK ANKRD13C UBP1 SS18L1 ZFP36L1 TFAM ZNF791 PAWR ARID2 ATG7 TMF1
Regulation of macromolecule biosynthetic process	0.017090142	11	CCNK ANKRD13C UBP1 SS18L1 ZFP36L1 TFAM ZNF791 PAWR ARID2 ATG7 TMF1
Regulation of cellular macromolecule biosynthetic process	0.026311351	10	CCNK UBP1 SS18L1 ZFP36L1 TFAM ZNF791 PAWR ARID2 ATG7 TMF1
Regulation of gene expression	0.026311351	10	CCNK UBP1 SS18L1 ZFP36L1 TFAM LRRC32 TMF1 ZNF791 PAWR ARID2
Regulation of nucleobase-containing compound metabolic process	0.026311351	10	CCNK UBP1 SS18L1 ZFP36L1 TFAM ZNF791 PAWR ARID2 ATG7 TMF1
Regulation of cellular biosynthetic process	0.026311351	10	CCNK UBP1 SS18L1 ZFP36L1 TFAM ZNF791 PAWR ARID2 ATG7 TMF1
Nucleic acid-templated transcription	0.026311351	9	CCNK UBP1 SS18L1 TFAM ZNF791 PAWR ZFP36L1 ARID2 TMF1
Regulation of nucleic acid-templated transcription	0.026311351	9	CCNK UBP1 SS18L1 TFAM ZNF791 PAWR ZFP36L1 ARID2 TMF1
Transcription, DNA-templated	0.026311351	9	CCNK UBP1 SS18L1 TFAM ZNF791 PAWR ZFP36L1 ARID2 TMF1
Regulation of transcription, DNA-templated	0.026311351	9	CCNK UBP1 SS18L1 TFAM ZNF791 PAWR ZFP36L1 ARID2 TMF1
Regulation of RNA biosynthetic process	0.026311351	9	CCNK UBP1 SS18L1 TFAM ZNF791 PAWR ZFP36L1 ARID2 TMF1
Organelle organization	0.026311351	9	NAA50 ATG7 ARID2 TFAM TMF1 PDCD6IP SS18L1 ATL3 PAWR
RNA biosynthetic process	0.026311351	9	CCNK UBP1 SS18L1 TFAM ZNF791 PAWR ZFP36L1 ARID2 TMF1
Regulation of RNA metabolic process	0.027906381	9	CCNK UBP1 SS18L1 ZFP36L1 TFAM ZNF791 PAWR ARID2 TMF1
Transcription by RNA polymerase II	0.026311351	8	CCNK UBP1 SS18L1 ZNF791 PAWR ZFP36L1 ARID2 TMF1
Regulation of transcription by RNA polymerase II	0.039387625	7	CCNK UBP1 SS18L1 ZNF791 PAWR ZFP36L1 ARID2
Organic substance transport	0.041746665	7	SLC16A7 ARL5A LRRC32 TMF1 PDCD6IP ZFP36L1 ATG7
Apoptotic process	0.039387625	6	PAWR ANKRD13C TMF1 PDCD6IP ZFP36L1 ATG7
Programmed cell death	0.042301013	6	PAWR ANKRD13C TMF1 PDCD6IP ZFP36L1 ATG7
Regulation of apoptotic process	0.049169362	5	PAWR ANKRD13C TMF1 ZFP36L1 ATG7
Endomembrane system organization	0.041746665	3	TMF1 ATL3 PDCD6IP
Regulation of lymphocyte activation	0.044943472	3	LRRC32 PAWR ZFP36L1
T cell activation	0.049545583	3	LRRC32 PAWR ZFP36L1
Regulation of cellular response to oxidative stress	0.026311351	2	PAWR ATG7
Regulation of oxidative stress-induced cell death	0.026311351	2	PAWR ATG7
Cell death in response to oxidative stress	0.026311351	2	PAWR ATG7
Negative regulation of T cell proliferation	0.026311351	2	LRRC32 PAWR
Regulation of response to oxidative stress	0.027906381	2	PAWR ATG7
Negative regulation of mononuclear cell proliferation	0.030401294	2	LRRC32 PAWR
Negative regulation of lymphocyte proliferation	0.030401294	2	LRRC32 PAWR
Negative regulation of leukocyte proliferation	0.031684653	2	LRRC32 PAWR
RegNetwork:RFX6	0.000458977	2	CCNK SS18L1
RegNetwork:GABPA	0.005380083	9	ZNF791 ZFP36L1 UBP1 TMF1 TFAM PDCD6IP NAA50 ATG7 ARID2
RegNetwork:E2F4	0.012531471	5	ZFP36L1 TFAM SS18L1 NAA50 ATL3
RegNetwork:SMARCA4	0.005595281	2	ARID2 TMF1
